# Monitoring *Fusarium* toxins from barley to malt: Targeted inoculation with *Fusarium culmorum*

**DOI:** 10.1007/s12550-024-00573-y

**Published:** 2024-12-20

**Authors:** Eva Maria Biehl, Sarah Schneidemann-Bostelmann, Felix Hoheneder, Stefan Asam, Ralph Hückelhoven, Michael Rychlik

**Affiliations:** 1 Chair of Analytical Food Chemistry, TUM School of Life Sciences, Technical University of Munich, Freising, Germany; 2 Chair of Phytopathology, TUM School of Life Sciences, Technical University of Munich, Freising, Germany

**Keywords:** *Fusarium culmorum*, *Fusarium* mycotoxins, Deoxynivalenol, Stable isotope dilution assay, LC–MS/MS analysis, Barley, Malt, Malting variations

## Abstract

Molds of the genus *Fusarium* infect nearly all types of grain, causing significant yield and quality losses. Many species of this genus produce mycotoxins, which pose significant risks to human and animal health. In beer production, the complex interaction between primary fungal metabolites and secondarily modified mycotoxins in barley, malt, and beer complicates the situation, highlighting the need for effective analytical methods to quickly and accurately monitor these toxins. We developed and validated a liquid chromatography-tandem mass spectrometry (LC–MS/MS) method to simultaneously analyze 14 *Fusarium* toxins, including modified forms (deoxynivalenol (DON), DON-3-glucoside, 3-acetyl-DON, 15-acetyl-DON, nivalenol, fusarenone X, HT-2 toxin, T-2 toxin, the enniatins A, A1, B, B1, beauvericin, and zearalenone) in barley and throughout the malting process. Stable isotope dilution assays (SIDAs) and matrix-matched calibration were used for quantification. A micro-malting setup was established to produce *Fusarium*-contaminated barley malt under reproducible conditions using targeted inoculation with *F. culmorum*. Mycotoxins were quantified throughout the malting process and compared to the content of fungal DNA. Further, the impact of various malting parameters was investigated, thus revealing that different malting scenarios exhibited different toxin enrichment patterns. We demonstrated that mycotoxin concentration and the ratio of DON to DON-3-glucoside changed throughout the malting processes, depending on fungal spore concentrations, germination temperature, and malting temperature. The study highlights the complexity of mycotoxin dynamics in malt production and the importance of optimized processing conditions to minimize toxin levels in final malt products.

**Supplementary Information:**

The online version contains supplementary material available at 10.1007/s12550-024-00573-y.

## Introduction

Molds of the genus *Fusarium* are globally prevalent and infect nearly all types of cereal grains. Due to their plant-pathogenic abilities, *Fusarium* species pose an economically significant problem because they lead to considerable yield losses in cereals (Thrane 2014). Moreover, infection with *Fusarium* often results in mycotoxin contamination, which can persist in stored grain for years, posing health risks to humans and animals (Desjardins 2006; Petronaitis et al. 2021). Previous studies (Müller & Schwadorf 1993; Wang et al. 1995; Placinta et al. 1999; Perkowski et al. 2007; Nathanail et al. 2015; Liu et al. 2016; Drakopoulos et al. 2021; Islam et al. 2021; Topi et al. 2021; Benešová et al. 2022; Del Palacio et al. 2023; Gozzi et al. 2024) have demonstrated that mycotoxin contamination, particularly with the *Fusarium* toxin deoxynivalenol (DON), a type B trichothecene, is a pervasive issue affecting wheat, barley, and maize globally. Research indicates that the extent of fungal infection and mycotoxin contaminations can vary significantly from year to year, influenced by weather conditions such as rainfall, air humidity, wind, and temperature (Windels 2000; Bai & Shaner 2004; Nathanail et al. 2015; Hofer et al. 2019; Hoheneder et al. 2022; Del Palacio et al. 2023).

In addition to primary fungal metabolites, modified mycotoxins resulting from interactions with the host plant are also relevant for food safety, given their potential exposure and presence in food and feed (Berthiller et al. 2013; Rychlik et al. 2014). However, the fundamentals and dependencies of the formation of these metabolites are only partially understood. The most studied and best-described modified *Fusarium* toxin is probably DON-3-glucoside (DON-3G), first described in 1986 as a metabolite of DON in wheat cell cultures (David Miller & Arnison 1986). In general, conjugated mycotoxins are often less toxic but can be reverted to their original aglycons in the digestive tract, thereby increasing systemic exposure toward these mycotoxins (Berthiller et al. 2011; Dall’Erta et al. 2013; Nagl et al. 2014). Therefore, conjugated mycotoxins should be regarded as equally harmful as their parent mycotoxins (EFSA Panel on Contaminants in the Food Chain 2014). For the frequently occurring and well-studied type B trichothecene DON and its derivatives, including 3-acetyl-DON (3-AcDON), 15-acetyl-DON (15-AcDON), and DON-3G, a group tolerable daily intake (TDI) of 1 µg/kg body weight per day was established in 2017 in the European Union (EFSA Panel on Contaminants in the Food Chain 2017). However, under the current European legislation, including the recently amended regulations, still, only DON is subject to maximum-level restrictions in cereals (European Commission 2024).

*Fusarium* infection of barley results in reduced kernel weights, decreased protein and starch quality, and altered protein composition. This poses a significant quality problem for the grain processing industry, such as malt and beer production, since the enzymatic characteristics of malting barley and cereals are significant and sometimes quality-limiting factors (Bechtel et al. 1985; Parry et al. 1995; Geißinger et al. 2022). In addition to the altered properties, the previously mentioned mycotoxin contamination of malting barley is particularly problematic, emphasizing the need for enhanced management of *Fusarium* infections in barley throughout the whole barley value chain (Hückelhoven et al. 2018). Various studies have shown that *Fusarium* mycotoxins originating from contaminated barley can pass through the entire process, from malting and brewing to the finished beer. Therefore, the consumption of beer may pose a risk to certain population groups. During the brewing process, the fate of trichothecenes (mainly type B) has been investigated predominantly (Schwarz et al. 1995; Wolf-Hall 2007; Lancova et al. 2008; Kostelanska et al. 2009, 2011b, 2011c; Maul et al. 2012; Inoue et al. 2013; Habler et al. 2016b, 2016c; Pascari et al. 2019; Prusova et al. 2022). However, the *Fusarium* peptide toxins beauvericin (BEA) and enniatins (ENN) have also been studied (Hu et al. 2014; Habler et al. 2016b, 2016c). According to the literature mentioned above, type B trichothecenes showed a significant decrease in DON content in the first step of the malting process due to “washing out” during soaking. After the subsequent germination, an increase in the DON concentration combined with a rapid increase in the amount of DON-3G could be demonstrated in the literature. The type B trichothecene concentrations were also hardly affected by following kilning, which can be attributed to the thermostability of DON and its acetylated derivatives up to 120 °C (Schwarz et al. 1995; Lancova et al. 2008; Vegi et al. 2011; Habler et al. 2016c; Pascari et al. 2019). According to Kostelanska et al. (2011b), kilning the malt at temperatures above 150 °C had a high potential to reduce the DON content, but this significantly changed the desired malt characteristics in terms of color and flavor (Kostelanska et al. 2011a, 2011b). However, previous studies reported varying transfer rates of type B trichothecenes into the finished beer during the subsequent brewing process (Schwarz et al. 1995; Lancova et al. 2008; Kostelanska et al. 2011c).

The existing toxin contamination data for barley, malt, and beer samples still need to be increased and require further research. Therefore, identifying *Fusarium* toxins and determining and controlling their levels are crucial, along with developing reliable analytical methods for rapidly and accurately monitoring them throughout the beer production chain. Previous studies have used field-inoculated material, but this approach also presents challenges. *Fusarium* toxins are not produced exclusively by a single species. Different toxins are produced by different species and strains, and several species co-occur on the grain. Hence, the effects of species competition on the final contamination of grain with various toxins are very likely and not under experimental control. Performing reproducible investigations of toxin concentration and distribution during malting is very challenging when using field-inoculated material. It is impossible to unequivocally attribute the toxin load solely to the species used for inoculation. The survival of the inoculum and the strength of the infection depend on weather conditions, further complicating the reproducibility of final grain contamination.

Therefore, in this study, we tried to create a reliable model system to study *Fusarium* infection and toxin distribution during malting. To produce malt, we used spring barley, a variety *Solist*, that we surface-disinfected to reduce natural contamination with molds. To create defined infected material, barley samples were inoculated explicitly with a strain of *Fusarium culmorum* (*F. culmorum*, *Fc*002), which was previously isolated from barley grains, before germination under reproducible laboratory conditions. Subsequently, a small-scale malting system with pre-defined inoculated starting material was developed and established, according to the Mitteleuropäische Analysenkommission (MEBAK) (MEBAK online). This allowed for precise tracking of toxin formation during the malting process depending on the amount of spores, temperature, and humidity. For toxin determination during the malting process, we developed and validated a multi-mycotoxin LC–MS/MS method for the analysis of 14 *Fusarium* toxins, including 3-AcDON, 15-AcDON, BEA, DON, DON-3G, ENN A, ENN A1, ENN B, ENN B1, fusarenone X (FUSX), HT-2 toxin (HT-2), nivalenol (NIV), T-2 toxin (T-2), and zearalenone (ZEN), in barley and malt.

The influence of different malting temperatures on toxin content and the effect of varying spore concentrations during malting were also investigated. Additionally, quantitative polymerase chain reaction (qPCR) was used to monitor fungal DNA content throughout the malting process and to correlate it with the obtained toxin data.

## Materials and methods

### Chemicals and analytical standards

Acetonitrile and methanol were purchased from Honeywell Riedel-de Haën (Seelze, Germany), formic acid (≥ 99%) from VWR (Darmstadt, Germany), and water from Th. Geyer (Renningen, Germany), all at least in analytical grade. Hydrogen peroxide solution (H_2_O_2_) (30%) and isopropanol were bought from VWR (Darmstadt, Germany) in technical grade and potato starch from Merck KGaA (Darmstadt, Germany). Unlabeled reference compounds (DON-3G, 15-AcDON, T-2) and some labeled standards ([^13^C_15_]-DON, [^13^C_17_]-3-AcDON, [^13^C_22_]-HT-2, and [^13^C_21_]-DON-3G) were purchased from Biopure (Tulln, Austria). DON, 3-AcDON, and FUSX were obtained from Coring System Diagnostix (Gernsheim, Germany). HT-2 and ZEN were purchased from Sigma-Aldrich (Missouri, USA). ENN A, ENN B, and NIV were obtained from Cayman Chemicals (Ann Arbor, USA), ENN A1 and ENN B1 from Enzo Life Sciences (Lörrach, Germany), and BEA from AnaSpec (San Jose, USA). [^15^N_3_]-BEA, [^15^N_3_]-ENN A1, and [^13^C_4_]-T-2 were synthesized in our laboratory as reported previously (Asam & Rychlik 2006; Hu & Rychlik 2012).

### Certified reference material

Certified reference material RM 1006460 (DON, ZEN, and ochratoxin A in wheat flour) was obtained from BioPure (Romer Labs, Tulln, Austria) and ERM®-BC600 (DON, ZEN and NIV in wheat flour) from the Federal Institute for Materials Research and Testing (Berlin, Germany).

### Barley raw material for malting

Malting experiments were conducted using spring barley cultivar *Solist* grains, harvested in 2019 and supplied by IREKS GmbH (Kulmbach, Germany). Barley grains were stored in jute bags in a dark place at 4 °C until use.

### Preparation of stock solutions

The analytes 3-AcDON, 15-AcDON, BEA, DON, ENN A, ENN A1, ENN B, ENN B1, FUSX, HT-2, NIV, ZEN, and the synthesized internal standards [^15^N_3_]-BEA, [^15^N_3_]-ENN A1, and [^13^C_4_]-T-2 underwent quantitative NMR in methanol-d_4_ to determine the concentration and purity of the analytes. In cases when the available amount of the compound was insufficient for recording a quantitative NMR spectrum, the certified specification of the manufacturer was considered as the concentration of the solution. This is applied to the following compounds: DON-3G, [^13^C_17_]-3-AcDON, [^13^C_15_]-DON, [^13^C_21_]-DON-3G, and [^13^C_22_]-HT-2. Stock solutions of unlabeled and labeled toxins were prepared in concentrations of 10 µg/mL in acetonitrile (DON, DON-3G, 3-AcDON, 15-AcDON, FUSX, HT-2, NIV, T-2, ZEN) and methanol (BEA, ENN A, ENN A1, ENN B, ENN B1) and further diluted to final concentrations of 1, 0.1, and 0.01 µg/L. All solutions were stored at 4 °C in the dark.

### Preparation of fungal inoculum

A single spore isolate of *F. culmorum* (*Fc*002), previously isolated from barley grain, was obtained from long-term storage (− 70 °C) glycerol stocks (culture collection, Chair of Phytopathology, Technical University of Munich) and further used for the preparation of fungal spore inoculum (Linkmeyer et al. 2013). Therefore, the isolates were cultivated on 1/4 strength PDA media (9.75 g/L potato dextrose agar, 11.75 g/L agar) for 14 days in an incubator (SAN-MLR-351H, SANYO, Munich, Germany) at 21 °C, 70% relative humidity (RH), and a 12 h light/dark cycle with additional UV-light (cool white, Osram Lumilux 840; UV-light, Philips TL-D BLB black light blue). Subsequently, mycelium and conidia were scraped off with a microscope slide and suspended in sterile tap water. Spores were separated from mycelium fragments by filtering through four layers of sterile gauze bandage placed in a Büchner funnel. Spore concentration was determined with a hemocytometer (Carl Roth GmbH & Co KG, Karlsruhe, Germany) and adjusted to the desired concentration (colony forming unit (CFU)/mL) by adding autoclaved tap water stepwise. The spore solution was stored in the dark at 4 °C for a maximum of 1 week.

### Grain surface disinfection

Barley grains were sanitized under sterile conditions according to Oliveira et al. (2012). Perforated stainless steel steeping boxes, pre-disinfected with 70% isopropanol, were used to disinfect 400 g of barley grains in 3 L of 10% H_2_O_2_ solution for 10 min with continuous stirring. The grains were washed for 5 min with 2 L of sterile tap water. Subsequently, the process was repeated under the same conditions for 5 min each. The disinfected grains were transferred to sterile stainless steel boxes and dried at room temperature for 24 h under sterile laminar airflow.

### Fungal inoculation of barley

Sanitized barley grains were inoculated with 2% (*v/w*) of the prepared *F. culmorum* spore suspension with a 10^5^ CFU/mL concentration. The grains were thoroughly mixed and incubated in a climate-controlled chamber (KMF 115, Binder GmbH, Tuttlingen, Germany) for 5 days at 25 °C and 96% RH to promote fungal proliferation. A control sample was also prepared by inoculating with sterile tap water. The grains were mixed every 12 h during the incubation period, and after 5 days, the grains were carefully homogenized, moisture contents were analyzed by freeze-drying for 48 h, and they were prepared for malting.

### Malting process

The malting processes were conducted on a laboratory scale according to the standard method described in the collection MEBAK R-110.45.008 published by the MEBAK. In detail, a 3-day steeping process was implemented (MEBAK 2010). On the first day, approximately 400 g of barley was steeped at 14 °C for 5 h, followed by a 19-h aeration period. On the second day, the barley underwent wet steeping for 4 h at 14 °C and then aerated for 20 h. On the third day, the steeping degree was adjusted to 44.5%, according to MEBAK, by spraying with water if necessary. Germination was then carried out for 3 days in a climate-controlled chamber set to 14 °C and 98% RH, with the green malt turned twice daily manually. The germinated barley grains were subsequently withered at 50 °C for 16 h, followed by kilning at 60 °C for 1 h, 70 °C for 1 h, and 80 °C for 5 h. After kilning, the rootlets were removed mechanically (Probenreiniger MLN, Pfeuffer GmbH, Kitzingen). All processes were conducted in biological triplicates per malting round.

### Variation of malting parameters

Additional lab-scale maltings were conducted to analyze the effects of different malting temperatures on toxin formation. For these experiments, the steeping and germination temperatures were either lowered from 14 to 10 °C or increased from 14 to 18 °C. The inoculations were carried out with a constant spore concentration of 10^5^ CFU/mL. Further lab-scale maltings were conducted to analyze the effects of spore load on toxin formation during the malting process. For this purpose, the *F. culmorum* spore concentration was increased from 1 × 10^5^ CFU/mL to 4 × 10^5^ CFU/mL and malted using standard conditions at 14 °C.

### Sampling

**Fig. 1:**
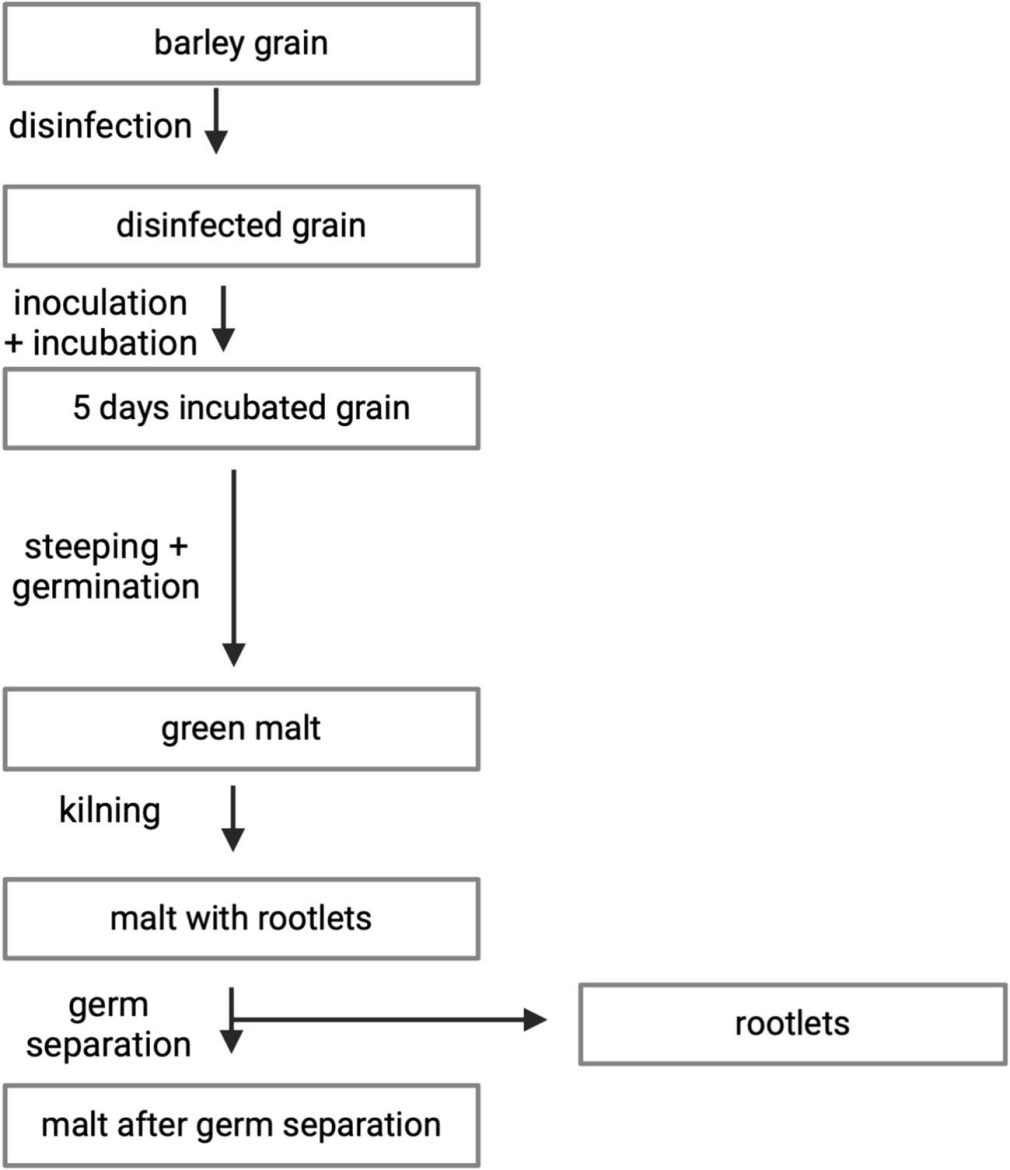
Scheme of the critical steps in the malting procedure (analyzed intermediates are shown in boxes)

Samples were taken at each critical step during the inoculation and malting process (see Fig. 1). Specifically, approximately 20–30 g sample material was taken from the barley grain, disinfected grain, 5 days incubated grain, green malt, malt with rootlets, malt after germ separation, and rootlets, respectively.

Green malt samples were freeze-dried for 48 h after sampling, their water contents were calculated, and the obtained toxin contents in the dried matter were corrected for the water content to report the final mycotoxin concentrations based on sample wet weights. Barley, disinfected grain, 5 days incubated barley, malt with rootlets, malt after germ separation, and rootlets were analyzed directly. All process samples were finely ground in a laboratory mill (Grindomix GM200, Retsch GmbH, Germany) to a fine powder and stored at 4 °C in the dark until further use.

### Quantification of genomic DNA with qPCR (barley and *F. culmorum*)

The genomic DNA extraction was carried out according to the European Reference Laboratory’s Joint Research Centre (2007) protocol for isolating maize DNA, with minor modifications described by Linkmeyer et al. (2013). The quantity of isolated genomic DNA was determined with a Nanodrop ND-1000 spectrophotometer (Thermo Scientific Inc, Wilmington, USA) and diluted with sterile double distilled water to a final concentration of 20 ng/μL and stored at 4 °C until further processing.

The quantification of fungal genomic DNA in the respective sample material was done using quantitative polymerase chain reaction (qPCR) as described by Nicolaisen et al. (2009). The PCR amplifications were performed in technical duplicates using an AriaMx Real-time PCR system (Agilent Technologies Inc., Santa Clara, USA) with *F. culmorum* (FculC561fwd/FculC614rev) and barley-specific primer pairs (Hor1f/Hor2r) (Nicolaisen et al. 2009). The absolute quantification of barley and *Fusarium* DNA was achieved by external standard calibration. Serial dilutions (100, 10, 1, 0.1, and 0.01 ng DNA) of pure fungal or barley DNA were prepared and included in the qPCR analysis. The DNA of *F. culmorum* (*Fc*) was normalized to the barley DNA content and expressed as pg *Fusarium* DNA per ng barley DNA.

### Sample preparation for mycotoxin analyses

Sample preparation for mycotoxin analysis was performed based on slight modifications of the method described by Habler and Rychlik (2016). In brief, 1 g of the finely ground sample was spiked with internal standards in amounts tailored to the anticipated concentrations of the respective analytes and taking into account the specified calibration ranges of the respective toxin. After evaporation of the solvent overnight, 10 mL of acetonitrile/water (84/16, v/v) was added and extracted using a laboratory shaker at 325 rpm for 2 h. The sample was filtered (folded filter 310, size 700 mm) (VWR, Darmstadt, Germany), and 4 mL of the filtrate was applied on a Bond Elut Mycotoxin cartridge (500 mg, 3 mL) (Agilent Technologies, Santa Clara, USA) placed on a vacuum manifold (Macherey–Nagel, Düren, Germany). The eluate was collected with the aid of a slight vacuum, evaporated to dryness using a nitrogen evaporator (Thermo Fisher Scientific, Waltham, USA) with the heat block set at 40 °C, reconstituted in 200 µL ACN/water (1/1, v/v), membrane filtered (PVDF, 0.22 µm), and subsequently centrifuged at 4 °C for 20 min at 3200 g. Until LC–MS/MS analysis, the supernatants were stored at 4 °C in the dark.

### LC–MS/MS analysis

Chromatographic separation was performed on a Shimadzu Nexera X2 UHPLC system (Shimadzu, Kyoto, Japan) using a Shim-pack Velox PFPP column (10 × 2.1 mm, 2.7 µm, Shimadzu, Duisburg, Germany) with a Shim-pack Velox PFPP guard-column (5 × 2.1 mm, Shimadzu, Duisburg, Germany) that was kept at 30 °C in the column oven. Electrospray ionization (ESI) was used for all analytes, but the MS was operated in the negative ESI mode for the analytes DON, DON-3G, NIV, and ZEN and in the positive ESI mode for the analytes 3-AcDON, 15-AcDON, BEA, ENN A, ENN A1, ENN B, ENN B1, FUSX, HT-2, and T-2. Therefore, two LC methods with different solvents were applied. For the negative ESI mode, the binary gradient system consisted of (A) water and (B) acetonitrile at a flow rate of 0.4 mL/min. The gradient was started and held at 10% B for 2 min, raised linearly from 10 to 99% B during the next 4 min, and held at 99% B for 1.5 min. Next, the mobile phase returned to 10% B during the next 1.5 min, and the system was equilibrated for 2 min before the next run.

The gradient for the positive ESI mode consisted of (A) 0.1% formic acid and (B) methanol with 0.1% formic acid at a flow rate of 0.4 mL/min and was started and held at 6% B for 2 min, raised linearly to 90% B within 14 min, raised further to 99% B during the next 2 min, and then maintained at 99% B for 1.5 min. Next, the mobile phase returned to 6% within 1.5 min, and the system was equilibrated for 2 min before the next run. The sample injection volume for both ESI modes was 5 µL with a coelution volume of water of 20 µL.

The LC system was connected to a Shimadzu 8050 triple quadrupole mass spectrometer (Shimadzu Corporation, Kyoto, Japan).

The ion source parameters for the negative mode were set as follows: interface temperature 340 °C, heat block temperature 430 °C, desolvation temperature 170 °C, interface voltage 4.5 kV, heating gas flow 10 L/min, drying gas flow 10 L/min, nebulizing gas flow 1.4 L/min, and collision-induced dissociation gas pressure 230 kPa.

The ion source parameters for the positive mode were set as follows: interface temperature 350 °C, heat block temperature 450 °C, desolvation temperature 150 °C, interface voltage 3 kV, heating gas flow 10 L/min, drying gas flow 10 L/min, nebulizing gas flow 3 L/min, and collision-induced dissociation gas pressure 265 kPa.

The mass spectrometer operated in scheduled multiple reaction monitoring (MRM) mode for MS/MS measurements under the conditions detailed in Table 1. Data acquisition and analysis were performed using LabSolutions Software (Shimadzu, Kyoto, Japan).

**Table 1:** List of fragment ions and retention times (Rt) of the analyzed *Fusarium* toxins and their corresponding optimized collision energies (CE) and voltages

Analyte	ESI + / −	Precursor ion *m/z*	Product ion *m/z*	Q1 pre-bias (V)	Collision energy (V)	Q3 pre-Bias (V)	Retention time (min)
NIV	−	311.20	281.20^a^ 138.20^b^	20 20	13 24	30 30	1.07
DON-3G	−	457.25	427.30^a^ 247.25^b^	12 12	19 20	28 24	1.28
[^13^C_21_]-DON-3G	−	478.25	447.30^a^ 261.25^b^	12 12	19 20	28 24	1.28
DON	−	295.30	265.20^a^ 247.20^b^	10 10	14 15	10 40	1.47
[^13^C_15_]-DON	−	310.30	279.20^a^ 261.20^b^	10 10	14 15	10 10	1.47
ZEN	−	317.15	175.10^a^ 131.05^b^	24 24	25 30	16 22	5.19
FUSX	+	355.10	175.20^a^ 137.20^b^	− 12 − 12	− 22 − 26	− 20 − 28	5.61
15-AcDON	+	339.25	261.20^a^ 321.25^b^	− 10 − 10	− 11 − 8	− 30 − 6	7.62
3-AcDON	+	339.10	231.25^a^ 175.20^b^	− 16 − 16	− 13 − 25	− 26 − 20	7.84
[^13^C_17_]-3-AcDON	+	356.10	245.25^a^ 186.00^b^	− 16 − 16	− 13 − 25	− 26 − 20	7.84
HT-2	+	447.15^c^	345.15^a^ 285.20^b^	− 22 − 22	− 19 − 21	− 18 − 20	11.4
[^13^C_22_]-HT-2	+	469.15^c^	362.15^a^ 300.20^b^	− 22 − 22	− 19 − 21	− 18 − 20	11.4
T-2	+	489.10^c^	245.15^a^ 387.15^b^	− 26 − 14	− 27 − 21	− 29 − 22	12.6
[^13^C_4_]-T-2	+	493.10^c^	245.15^a^ 391.15^b^	− 26 − 14	− 27 − 21	− 29 − 22	12.6
ENN B	+	640.75	196.25^a^ 214.25^b^	− 18 − 18	− 25 − 25	− 22 − 16	15.8
ENN B1	+	654.30	196.25^a^ 210.25^b^	− 34 − 32	− 26 − 24	− 23 − 24	16.0
ENN A1	+	668.70	210.25^a^ 100.20^b^	− 18 − 18	− 24 − 60	− 16 − 20	16.2
ENN A	+	682.70	210.20^a^ 100.15^b^	− 12 − 12	− 25 − 55	− 16 − 20	16.4
[^15^N_3_]-ENN A1	+	671.70	211.25^a^ 101.20^b^	− 18 − 18	− 24 − 60	− 16 − 20	16.2
BEA	+	784.55^c^	134.20^a^ 244.25^b^	− 22 − 22	− 59 − 32	− 26 − 28	16.3
[^15^N_3_]-BEA	+	787.55^c^	135.20^a^ 245.25^b^	− 22 − 22	− 59 − 32	− 26 − 28	16.3

^a^Quantifier

^b^Qualifier

^c^Sodium adduct [M + Na]^+^

### Calibration and quantitation

Quantifying toxins in all samples was performed using specific response functions or respective matrix calibration functions. For toxins that were quantified using SIDA (DON, DON-3G, T-2, HT-2, 3-AcDON, ENN A1, BEA), constant amounts of the stable isotope-labeled standards (S) were mixed with varying amounts of their non-labeled forms (A) to obtain molar ratios in the range n(A)/n(S) from 0.01–100 (1:100, 1:50, 1:10, 1:5, 1:2, 1:1, 2:1, 5:1, 10.1, 50:1, 100:1). Response curves were obtained using linear regression by plotting peak area ratios [A(A)/A(S)] against molar ratios [n(A)/n(S)]. For some analytes, no respective isotopically labeled standards were applied: [^13^C_17_]-3-AcDON was used as the internal standard for 15-AcDON, and ENN A, ENN B, and ENN B1 were determined using [^15^N_3_]-ENN A1 as the internal standard. Response curves were recorded in the same manner as described for the SIDA.

Matrix-matched calibration was performed using potato starch as a blank matrix for analytes without internal standards (NIV, ZEA, and FUSX). No real barley sample was completely devoid of mycotoxins and could serve as a blank matrix for matrix calibration, so we used potato starch instead which has been found to be free from mycotoxins by previous analyses. Therefore, 11 matrix calibration points were prepared for each toxin, ranging from 7.5–750 µg/kg for NIV, 0.3–45 µg/kg for ZEN, and 5–500 µg/kg for FUSX. After solvent evaporation, the above-described sample preparation was performed.

Following the LC–MS/MS measurements, matrix-matched calibration curves were obtained by plotting peak areas against concentrations and performing linear regression. Three to four matrix calibration points were prepared with each sample batch to account for daily variabilities in the LC–MS/MS system’s intensity. These calibration points were then used to check for the validity of the previously established matrix calibration curves.

Linearity was confirmed for all analytes by applying Mandel’s fitting test (Mandel 1964).

### Method validation

#### LODs and LOQs

Limits of detection (LODs) and limits of quantification (LOQs) were determined as described by Vogelgesang and Hädrich (1998). Therefore, a *Fusarium* toxin-free potato starch was used as a blank matrix, spiked with the analytes in four different concentration levels, each in triplicates (see Table S1 in the supplementary material for detailed information). If applicable, internal standards were added in constant concentrations, and samples were further prepared for LC–MS/MS measurements following the described sample workup procedure.

#### Precision

The blank matrix (potato starch) was spiked in triplicate with all analytes and internal standards if applicable (see Table S1 in the supplementary material for detailed information). It was then subjected to sample preparation and used for precision measurements for *intra-day* (*n* = 3) and *inter-day* (*n* = 9, triplicate measurement every week within 3 weeks). For *inter-injection* precisions, analyte mixtures were injected in the LC–MS/MS system ten times in a row.

#### Recovery

Blank samples were spiked in triplicate with three different concentrations of each analyte and internal standards if applicable (see Table S1 in the supplementary material for detailed information) and analyzed as described previously. Recoveries were calculated using the ratio of detected and spiked toxin contents.

#### Accuracy

Two reference materials (containing 825 ± 248 µg/kg DON respectively 102 ± 11 µg/kg DON in wheat flour) were analyzed after the addition of internal standards in triplicates as described previously.

## Results and discussion

### LC–MS/MS

During the development of the method, it was necessary to find compromises in the measurement parameters to enable the most sensitive determination possible for all the analytes to be quantified. To increase sensitivity, we decided to measure DON, DON-3G, NIV, and ZEN in the negative ESI mode as [M-H]^−^ ions. The other ten analytes (3-AcDON, 15-AcDON, BEA, ENN A, ENN A1, ENN B, ENN B1, FUSX, HT-2, and T-2) were analyzed in the positive mode. The acetylated derivatives of DON, FUSX, and the ENNs were measured as [M + H]^+^ ions, whereas sodium adducts [M + Na]^+^ could be measured most intensively and reproducibly for the type A trichothecenes and BEA. To achieve these optimal MS conditions for all analytes, it was necessary to use the adapted composition of mobile phases, and each sample underwent two chromatographic runs: one in positive ESI mode and one in negative ESI mode.

Using the Shim-pack Velox PFPP column (Shimadzu, Duisburg, Germany), it was possible to chromatographically separate DON and DON-3G as well as the constitutional isomers 3-AcDON and 15-AcDON. The separation of DON and DON-3G is important because DON-3G has a tendency of in-source fragmentation, making it indistinguishable from DON without chromatographic separation, although this effect is more pronounced in the positive ESI mode. The separation of the isomers 3- and 15-AcDON is necessary for simultaneous quantification, although there are reports about the differentiation of the two analytes using differences in the fragmentation pattern in the mass spectrometer (Berthiller et al. 2005). The analytes BEA and ENN A1 coeluted at a similar retention time but could be clearly distinguished by their different molecular masses and fragmentation patterns (see Table 1).

### Sample preparation

The sample cleanup was based on a previously published method by Habler and Rychlik (2016) to analyze *Fusarium* toxins in grains, with some modifications. Unlike the original procedure, internal standards were added before the sample extraction to compensate for potential extraction losses. Additionally, DON-3G was quantified for the first time using the corresponding fully isotopically labeled standard ([^13^C_21_]-DON-3G) instead of matrix-matched calibration or partially isotopically labeled [^13^C_6_]-DON-3G (Habler et al. 2016a). Using a nitrogen evaporation unit instead of a rotary evaporator significantly facilitated sample preparation by simultaneously removing the solvent from up to 72 samples. An additional centrifugation step at 4 °C after microfiltration was implemented to remove particles, particularly in samples with high matrix loads (green malt, rootlets). This helped prevent clogging of the HPLC column as well as contamination of the mass spectrometer.

### Calibration and quantitation

Calibration curves were obtained using linear regression and were checked with Mandel’s fitting test for their linearity (Mandel 1964). The response curves showed linearity for DON, DON-3G, and T-2 within the molar ratios 0.01–100; for 3-AcDON, 15-AcDON, ENN A, ENN A1, ENN B, and HT-2 within 0.01–50; and for BEA and ENN B1 within 0.01–10. A wide range of linearity for the matrix-matched calibration was determined for FUSX between 15 and 150 µg/kg, NIV between 7.5 and 750 µg/kg, and ZEN between 0.3 and 22.5 µg/kg.

### Validation

Method validation was carried out according to Vogelgesang and Hädrich (1998). A detailed summary of the results is presented in Table 2. To determine the LODs and LOQs, an analyte-free starch sample was spiked in triplicate at four different concentration levels. For analytes quantified with internal standard, LODs ranged from 0.01 µg/kg (BEA, ENN B, and ENN B1) to 1.85 µg/kg (DON-3G), and LOQs ranged from 0.03 µg/kg (ENN B) to 5.71 µg/kg (DON-3G). The type B trichothecenes FUSX and NIV quantified through matrix-matched calibration exhibited LODs and LOQs of 2.93 µg/kg and 10.8 µg/kg (FUSX) as well as 1.77 µg/kg and 5.25 µg/kg (NIV). Thus, FUSX had the highest LOD and LOQ among all the identified toxins. ZEN could be quantified with an LOD of 0.14 µg/kg and an LOQ of 0.45 µg/kg using matrix-matched calibration. The determined LODs and LOQs are comparable to or lower than the values reported in the literature (Sulyok et al. 2006; Malachová et al. 2014; Nathanail et al. 2015; Decleer et al. 2016; Habler & Rychlik 2016; Benešová et al. 2022; Karlsson et al. 2023). The lower LOD and LOQ of 0.03 µg/kg respective 0.09 µg/kg for DON achieved by Tolosa et al. (2017) can be attributed to a fourfold sample weight, the use of a high-resolution mass spectrometer, and the estimation of the limits through signal-to-noise ratio (*S/N*) evaluation. However, as DON was one of the predominant toxins in our study, our values were more than sufficient.

**Table 2:** Validation data including limits of detection (LODs), limits of quantitation (LOQs), precision (RSD), and recoveries (3 different concentration levels) for 14 *Fusarium* toxins in starch. Recovery values of each spiking level were calculated as the mean value of three replicates and three injections

Analyte	Analysis	LOD (µg/kg)	LOQ (µg/kg)	Precision (RSD) (%)			Recovery (%)		
				Inter-injection (*n* = 10)	Intra-day (*n* = 3)	Inter-day (*n* = 9)	Level 1	Level 2	Level 3
DON	SIDA	0.60	1.77	5	1	3	90 ± 1	96 ± 2	95 ± 1
DON-3G	SIDA	1.85	5.71	6	3	6	99 ± 3	96 ± 2	114 ± 3
3-AcDON	SIDA	0.11	0.45	5	0.3	5	96 ± 1	88 ± 2	84 ± 1
15-AcDON	SIDA	0.63	2.56	6	2	7	101 ± 2	100 ± 4	93 ± 1
HT-2	SIDA	0.02	0.09	3	3	1	96 ± 3	101 ± 1	103 ± 2
T-2	SIDA	0.02	0.05	3	3	2	99 ± 3	100 ± 2	103 ± 2
ENN A	IS	0.02	0.05	7	2	6	95 ± 2	82 ± 7	87 ± 1
ENN A1	SIDA	0.05	0.21	5	2	6	100 ± 2	98 ± 6	103 ± 5
ENN B	IS	0.01	0.03	5	4	5	106 ± 4	101 ± 0.2	102 ± 3
ENN B1	IS	0.01	0.04	5	2	9	99 ± 2	99 ± 6	102 ± 2
BEA	SIDA	0.01	0.06	2	2	5	104 ± 4	106 ± 2	86 ± 4
NIV	MMC	1.77	5.25	4	6	7	111 ± 0.1	109 ± 6	114 ± 10
ZEN	MMC	0.14	0.45	2	3	10	109 ± 8	89 ± 9	93 ± 8
FUSX	MMC	2.93	10.8	2	2	3	98 ± 7	101 ± 7	92 ± 5

*RSD* relative standard deviation, *SIDA* stable isotope dilution assay, *IS* internal standard quantification, *MMC* matrix-matched calibration

The *Fusarium* toxins were spiked at three different concentration levels in triplicates to an analyte-free matrix (potato starch) to determine the recoveries. The spiking levels were chosen to reflect the toxin concentrations typically found in naturally contaminated materials, allowing for monitoring concentration-dependent differences in recoveries. High contamination levels can occur, particularly for DON and DON-3G, and, therefore, were also reflected in the recovery samples. Recovery rates were 82% (ENN A) to 114% (DON-3G, NIV) and thus fell into the acceptable range of 70 to 120% (see Table 2).

The *intra-day*, *inter-day*, and *inter-injection* precisions were assessed by calculating each analyte’s relative standard deviation (RSD) after a specified number of repeated measurements. These are shown in Table 2. For *intra-day* precisions (*n* = 3), a starch sample was spiked once, while for *inter-day* precisions (*n* = 9), one spiked sample was prepared every week within 3 weeks, respectively. RSD values determined were less than or equal to 10%, which shows the good precision of the method used. To determine *inter-injection* precisions, analyte mixtures were injected ten times in a row in the LC–MS/MS system, resulting in RSDs of 2–7%, confirming the stability of the used LC–MS/MS system.

The trueness of the method was confirmed for DON by analyzing two commercially available reference materials. The reference materials used consisted of wheat flour containing 825 ± 248 µg/kg and 102 ± 11 µg/kg DON. Our analysis of DON resulted in contents of 745 ± 5.7 µg/kg and 106 ± 5.1 µg/kg, respectively, which were, therefore, within the content range specified by the manufacturer with deviations of − 10.3% and 3.9%, respectively. The validation results were, hence, satisfactory.

### Analyzing the raw material

The fate of the *Fusarium* toxins during the malting process was monitored using the developed LC–MS/MS method, including quantification with SIDA, internal standards, and matrix-matched calibration curves.

Initially, the barley variety used (*Solist*), a two-row spring barley primarily bred for brewing beer, was analyzed for its mycotoxin content. The barley contained 25.2 ± 2.98 µg/kg DON, 9.69 ± 0.35 µg/kg DON-3G, and 3.86 ± 0.16 µg/kg HT-2. DON and DON-3G concentrations were relatively low compared to the trichothecene contaminations reported in the literature. However, a DON/DON-3G ratio greater than 1 is typical for raw grains (Zachariasova et al. 2010; Kostelanska et al. 2011b; Nathanail et al. 2015; Piacentini et al. 2019). Among the analyzed type A trichothecenes, only HT-2 was found at a low level, which is also lower than the average concentrations reported by Nathanail et al. (2015) and Kostelanska et al. (2011b). 3-AcDON, 15-AcDON, FUSX, NIV, T-2, and ZEN were not detected in the barley. The depsipeptides were quantified as 0.08 ± 0.01 µg/kg ENN A, 0.43 ± 0.01 µg/kg ENN A1, 3.51 ± 0.14 µg/kg ENN B, 1.35 ± 0.05 µg/kg ENN B1, and 1.83 ± 0.12 µg/kg BEA. As already observed by Hu et al. (2014), Vaclavikova et al. (2013), Uhlig et al. (2006), and Habler et al. (2016c), the ENNs were determined in decreasing concentration order from ENN B, ENN B1, ENN A1, to ENN A. However, the maximum levels found here were far below the contamination levels of up to 240 µg/kg ENN B found by Habler et al. (2016c) and up to 1 mg/kg ENN B by Vaclavikova et al. (2013).

In addition to toxin analysis, the raw barley was examined for its *F. culmorum* DNA content using qPCR. The barley contained 0.0013 pg *F. culmorum* DNA/ng barley DNA, lower than the fungal contaminations determined by Habler et al. (2016c) for naturally infected barley. To summarize, the present barley was very low in toxins and was considered suitable for the following malting trials, therefore.

### Development and establishment of a small-scale malting system

A small-scale malting system was developed and established to monitor the toxin levels during the malting process. The barley was malted in this system according to the standard small-scale malting scheme from MEBAK, following thorough surface sterilization and subsequent inoculation. The kilning of the green malt was also conducted according to a standardized procedure.

The barley grains were first subjected to surface disinfection to remove the natural microflora. Bacteria and molds could influence future malting processes, cause cross-contamination with the subsequently inoculated *F.* *culmorum* spores, and thus falsify the analyses. The surface sterilization had no negative impact on the germination capacity of the barley grains, as the germination capacity of the barley remained consistently high after surface disinfection, still exceeding 98%, similar to Oliveira et al. (2012).

### Inoculation of barley with *F.culmorum*

*F. culmorum* was selected as a *Fusarium* species for targeted inoculation, using exclusively a single isolate from barley (*Fc*002) for all malting trials (Linkmeyer et al. 2013). Optimal climatic conditions for growth were chosen at 25 °C and 96% RH for 5 days (Brennan et al. 2003). For the inoculation, *F. culmorum* spores were applied to the disinfected barley grains immediately after surface sterilization under a sterile hood. Fungal growth was demonstrated by an increase in detected *F. culmorum* DNA. Subsequently, the material was malted according to the MEBAK small-scale malting scheme.

### The fate of *Fusarium* toxins during malting

#### Targeted inoculation with *F. culmorum*

To monitor the progression of toxins during the malting process, a barley sample was inoculated with a defined spore concentration of *F. culmorum* (10^5^ CFU/mL) and incubated for 5 days. In parallel, a control barley sample was prepared with sterile tap water instead of the spore solution. To conduct defined, reproducible experiments, continuous monitoring of the moisture content of the germinating material through gravimetric measurements and the control of temperature and humidity conditions in the constant climate chamber used was essential. Therefore, the barley samples were homogenized twice daily throughout the malting process. At crucial steps of the developed malting process (see Fig. 1), samples were taken and analyzed for their toxin and *F. culmorum* DNA content. Due to the increased moisture content and limited storage capacity, the green malt was immediately freeze-dried after sampling. After inoculation with *F. culmorum* spore solution (10^5^ CFU/mL), no visually detectable fungal growth could be observed on the malting material throughout the entire malting process. This was satisfactory, as the intention was to produce contaminated material that was not immediately visibly “infected.”

#### Analysis of mycotoxins

All samples were analyzed for their analyte content of all 14 *Fusarium* toxins using the developed LC–MS/MS method and are reported in µg/kg fresh weight. Since the focus of the experiments was on type B trichothecenes due to the *F. culmorum* strain used, these toxins are explained and discussed in the following. Nevertheless, the other toxins were analyzed, and the complete listing of all toxin levels during the malting processes can be found in the supplementary material (Tables S2–S6). Only FUSX, NIV, T-2, and ZEN were not detected in any samples of the malting processes and are not further mentioned.

#### Malting of infected barley

**Fig. 2:**
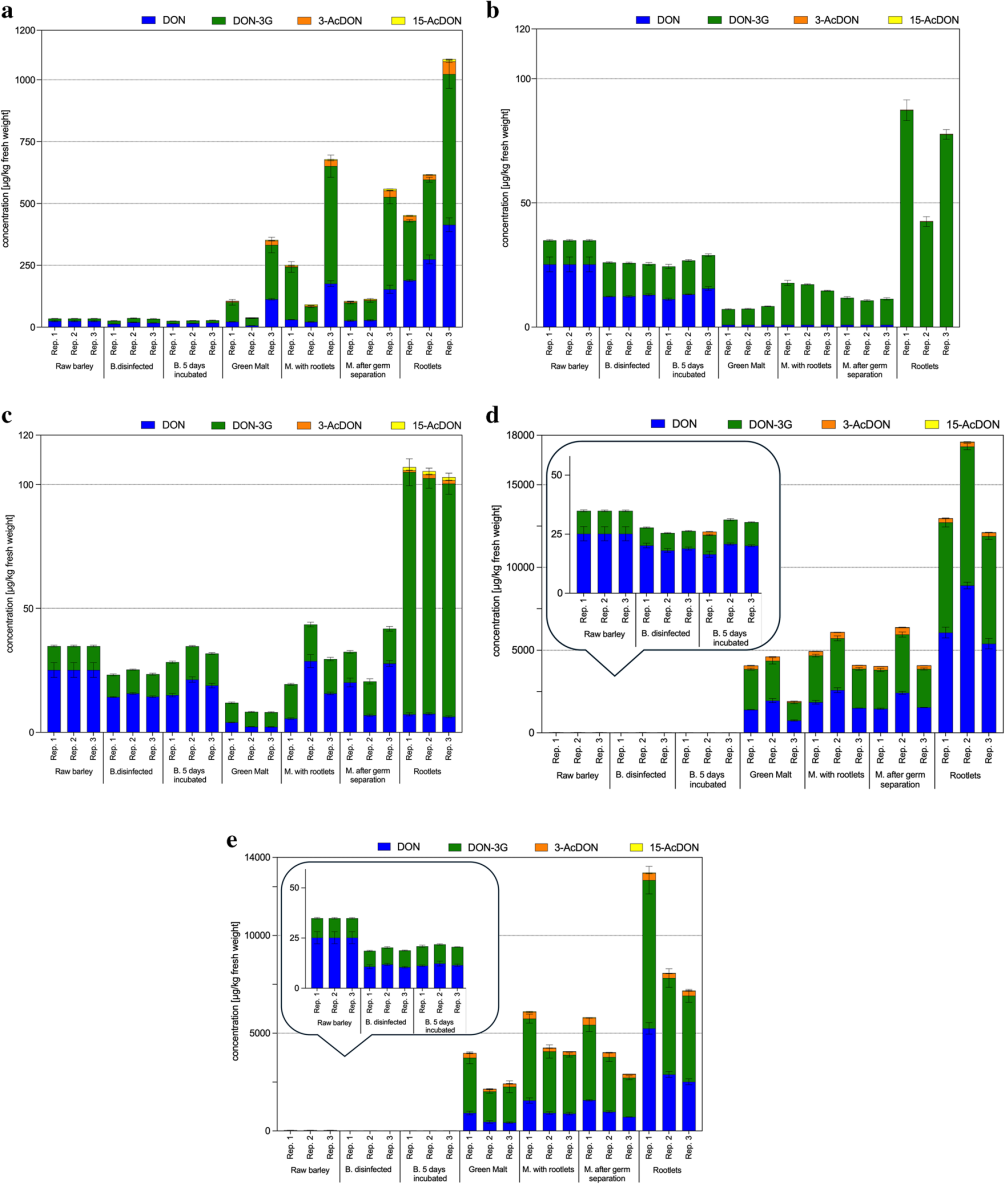
**a** Monitoring of type B trichothecenes DON, DON-3G, 3-AcDON, and 15-AcDON during the malting process following targeted inoculation with *F. culmorum* (10^5^ CFU/mL) at 14 °C malting temperature. **b** Monitoring of type B trichothecenes DON, DON-3G, 3-AcDON, and 15-AcDON during the control malting trial (inoculation with sterile tap water) at 14 °C malting temperature. **c** Monitoring of type B trichothecenes DON, DON-3G, 3-AcDON, and 15-AcDON during the malting process following targeted inoculation with *F. culmorum* (10^5^ CFU/mL) at 10 °C malting temperature. **d** Monitoring of type B trichothecenes DON, DON-3G, 3-AcDON, and 15-AcDON during the malting process following targeted inoculation with *F. culmorum* (10^5^ CFU/mL) at 18 °C malting temperature. **e** Monitoring of type B trichothecenes DON, DON-3G, 3-AcDON, and 15-AcDON during the malting process following targeted inoculation with *F. culmorum* (4 × 10^5^ CFU/mL) at 14 °C malting temperature. Data are shown for each of the three independent biological replicates (1–3) and are presented in µg/kg ± SD fresh weight. All values are given as the mean of triplicate determinations and duplicate injections. To analyze and represent values below LOQ, we used 1/2 × LOQ in graphical representations. Values below the LOD were recorded as 0 µg/kg. B = barley, M = malt

Figure 2a shows the levels of 3-AcDON, 15-AcDON, DON, and DON-3G during the malting process of the previously inoculated barley. The malting was conducted in biological triplicates. A summary of the determined concentrations as means ± standard deviation (SD) is provided in Table 3. The initial raw barley material, which was contaminated with 25.2 ± 2.98 µg/kg DON and 9.69 ± 0.35 µg/kg DON-3G, resulting in a DON/DON-3G ratio of 2.6, showed a reduction in DON content of approximately 34% after surface sterilization. In contrast, the DON-3G concentration increased by an average of 60%. The 5-day incubation at 25 °C had little impact on the DON concentrations, which remained unchanged. The DON-3G levels decreased, and an average of 62% of the initial DON-3G content was recovered before malting. During the subsequent steeping and germination phases, differences in DON content were observed in the green malt of the experimental setups. DON levels of 6.57–113 µg/kg were determined. After kilning, all samples showed an increase in DON concentration. Calculating the toxin content of the samples to dry weight reveals an increase in toxin concentration not attributable to water loss, suggesting new formation during the kilning process. After removing the highly contaminated rootlets, DON levels of 26.5–153 µg/kg were detected in malt. Thus, a 105–607% increase of the initial DON content in the raw material was quantified in the final kilned malt. The rootlets had the highest DON levels, ranging from 189–414 µg/kg. The modified toxin DON-3G showed a significant increase in concentration in the green malt after steeping and subsequent germination, up to 2260% compared to the initial value in raw material. After kilning, a further increase in concentration was observed. After removing the highly contaminated rootlets, the final cleaned kilned malt contained 72.4–373 µg/kg DON-3G, representing an increase of 747–3847% compared to the initial DON-3G concentration in the raw material. Despite strong differences in individual concentrations, the DON/DON-3G ratio changed from 2.6 to 0.3 throughout the experiment, showing similar tendencies between the biological triplicates. The DON/DON-3G ratio was approximately 0.8 in the rootlet byproduct. The acetylated DON derivative 3-AcDON could only be detected from the germination process onward, with levels in green malt ranging from 1.21–17.5 µg/kg. After kilning and removing the rootlets, 3-AcDON levels of 2.97–25.9 µg/kg were found in the final kilned malt. The separated rootlet byproduct was also highly contaminated with 3-AcDON, with levels up to 51.0 µg/kg. Furthermore, 15-AcDON was also detected starting from the green malt samples, but its concentrations were below the LOD. After kilning and cleaning, 15-AcDON contents in the kilned malt ranged from 2.90–7.11 µg/kg. The levels of 3-AcDON were generally up to 10 times higher than those of its constitutional isomer 15-AcDON.

**Table 3:** Mycotoxin concentrations of DON, DON-3G, 3-AcDON, and 15-AcDON, as well as fungal *Fc* DNA contents in the conducted malting trials. Data are presented in µg/kg ± SD as a mean of biological triplicates, triplicate determinations, and duplicate injections. The ratio of DON/DON-3G is calculated using the mean values

Spore amount	Malting temperatures	Sample type	DON (µg/kg)	DON-3G (µg/kg)	3-AcDON (µg/kg)	15-AcDON (µg/kg)	Ratio DON/DON-3G	*Fc* DNA pg/ng barley DNA
10^5^ CFU/mL	14 °C	B. raw	25.2 ± 2.98	9.69 ± 0.35	n. d.	n. d.	2.60	0.0013
		B. surface disinfected	16.6 ± 3.36	15.5 ± 2.08	n. d.	n. d.	1.07	0.0015 ± 0.0012
		B. 5 days incubated	15.6 ± 1.43	11.0 ± 0.17	n. d.	n. d.	1.43	0.0366 ± 0.0353
		Green malt	47.2 ± 57.6	109 ± 97.9	7.61 ± 8.68	< LOQ	0.43	0.1856 ± 0.0945
		M. with rootlets	75.8 ± 86.5	250 ± 209	10.5 ± 12.0	3.92 ± 0.96	0.30	0.3491 ± 0.2353
		M. after germ separation	69.1 ± 72.5	175 ± 171	10.8 ± 29.3	4.35 ± 2.40	0.39	0.2046 ± 0.2319
		Rootlets	292 ± 113	391 ± 193	29.3 ± 18.7	5.21 ± 3.77	0.75	0.4807 ± 0.2592
– (control)	14 °C	B. raw	25.2 ± 2.98	9.69 ± 0.35	n. d.	n. d.	2.60	0.0013
		B. surface disinfected	12.6 ± 0.37	13.2 ± 0.67	n. d.	n. d.	0.96	0.0028 ± 0.0024
		B. 5 days incubated	13.3 ± 2.17	13.4 ± 0.22	n. d.	n. d.	0.99	0.0013 ± 0.0004
		Green malt	< LOQ	6.77 ± 0.63	n. d.	n. d.	-	0.0040 ± 0.0053
		M. with rootlets	< LOQ	15.7 ± 1.66	n. d.	n. d.	-	0.0006 ± 0.0007
		M. after germ separation	< LOQ	10.4 ± 0.53	n. d.	n. d.	-	0.0007 ± 0.0006
		Rootlets	n. d.	69.1 ± 23.6	< LOQ	n. d.	-	0.0030 ± 0.0016
10^5^ CFU/mL	10 °C	B. raw	25.2 ± 2.98	9.69 ± 0.35	n. d.	n. d.	2.60	0.0013
		B. surface disinfected	14.8 ± 0.79	9.21 ± 0.38	n. d.	n. d.	1.61	0.0002 ± 0.0004
		B. 5 days incubated	18.5 ± 3.18	13.2 ± 0.27	n. d.	n. d.	1.39	0.0069 ± 0.0032
		Green malt	2.85 ± 1.03	6.61 ± 1.15	n. d.	n. d.	0.43	0.0568 ± 0.0208
		M. with rootlets	16.7 ± 11.6	14.2 ± 0.52	n. d.	n. d.	1.18	0.0771 ± 0.0211
		M. after germ separation	18.3 ± 10.6	13.4 ± 0.92	n. d.	n. d.	1.37	0.0617 ± 0.0078
		Rootlets	7.04 ± 0.64	95.6 ± 1.89	1.21 ± 0.40	< LOQ	0.07	0.2950 ± 0.1079
10^5^ CFU/mL	18 °C	B. raw	25.2 ± 2.98	9.69 ± 0.35	n. d.	n. d.	2.60	0.0013
		B. surface disinfected	19.1 ± 1.07	7.45 ± 0.11	n. d.	n. d.	2.56	0.0019 ± 0.0007
		B. 5 days incubated	19.2 ± 2.36	9.48 ± 1.11	n. d.	n. d.	2.02	0.0090 + 0.0111
		Green malt	1367 ± 601	1987 ± 773	177 ± 99.2	4.68 ± 2.15	0.69	16.4441 ± 5.6959
		M. with rootlets	1981 ± 553	2779 ± 383	278 ± 75.3	12.1 ± 3.18	0.71	14.7363 ± 4.4745
		M. after germ separation	1809 ± 531	2734 ± 691	282 ± 120	11.9 ± 6.99	0.66	7.5440 ± 0.4794
		Rootlets	6794 ± 1861	7182 ± 1064	238 ± 26.7	25.4 ± 5.33	0.95	112.61 ± 22.2505
4 × 10^5^ CFU/mL	14 °C	B. raw	25.2 ± 2.98	9.69 ± 0.35	n. d.	n. d.	2.60	0.0013
		B. surface disinfected	11.1 ± 0.73	8.20 ± 0.27	n. d.	n. d.	1.35	0.6062 ± 1.0497
		B. 5 days incubated	11.7 ± 0.57	9.45 ± 0.29	n. d	n. d.	1.24	44.3611 ± 8.1058
		Green malt	604 ± 274	2071 ± 667	168 ± 54.3	8.96 ± 1.76	0.29	43.6371 ± 4.3455
		M. with rootlets	1123 ± 377	3446 ± 648	226 ± 100	21.3 ± 4.38	0.33	31.4159 ± 4.0176
		M. after germ separation	1088 ± 439	2888 ± 927	255 ± 92.3	23.0 ± 5.81	0.38	35.2512 ± 4.7762
		Rootlets	3552 ± 1481	5639 ± 1706	278 ± 68.9	26.8 ± 2.39	0.63	5.1018 ± 0.8116

*B* barley, *M* malt, *n. d.* not detected, *LOQ* limit of quantification

#### Control

For the control sample and the inoculation with sterile tap water instead of spore solution, the 5-day incubation time at 25 °C showed no significant impact on the trichothecene toxin concentrations, which remained unchanged. The subsequent malting process, including steeping and germination phases, led to a significant reduction in DON content, which fell below the LOD in all green malt and malt samples, and DON was not found in the rootlets. In contrast, DON-3G was detected in green malt at an average concentration of 6.77 ± 0.63 µg/kg and in the uncleaned malt after kilning at 15.7 ± 1.66 µg/kg. After rootlet removal, the final cleaned malt contained an average of 10.4 ± 0.53 µg/kg DON-3G. The rootlet fraction, where 3-AcDON was detected for the first time but below the LOD, showed considerable variation in DON-3G concentrations between the three biological replicates, ranging from 42.5 to 87.3 µg/kg, with fluctuations up to 34%. The fate of the type B trichothecenes is shown in Fig. 2b, and their concentrations are listed in Table 3.

### Variation of malting parameters

#### Variation of malting temperatures

The germination temperature is a previously unexplored parameter in toxin formation during barley malting. According to MEBAK guidelines, micro-malting should be conducted at 14 °C, which refers to both the steeping water and the germination temperature. To investigate the influence of temperature, variations below (10 °C) and above (18 °C) the standard conditions were chosen. The spore concentration was consistently maintained at 10^5^CFU/mL. As previously observed, no mold infection was visually detectable during the malting processes.

Lowering the malting temperature to 10 °C resulted in visibly slower germination. The development of the germination tips was delayed, and in the green malt samples, rootlet formation was significantly less pronounced compared to malting at 14 °C. The toxin analysis of trichothecenes revealed similar values in surface-disinfected and incubated samples as observed in previously conducted maltings, as these steps were not affected by the reduced temperature. However, the following process samples showed significantly lower toxin levels than those of malting at 14 °C. After germination, the green malt samples contained DON levels ranging from 2.24 to 4.03 µg/kg and DON-3G from 5.89 to 7.93 µg/kg. This represented, on average, 11% of the original DON and 68% of the original DON-3G concentrations in the raw material. After kilning, the final malt showed an increase in levels to 6.93–20.2 µg/kg for DON and 12.3–14.1 µg/kg for DON-3G, corresponding to 73% and 138% of the initial values, respectively. The removed rootlets contained 6.32–7.51 µg/kg DON and 94.0–97.7 µg/kg DON-3G. The ratio of the aglycone to its modified form shifted during the malting process, from a ratio of 0.4 in the green malt to 1.4 in the final kilned malt. 3-AcDON was detected and quantified only in the rootlets, with concentrations ranging from 0.75 to 1.51 µg/kg, while the concentration of 15-AcDON remained below the LOD (see Fig. 2c, Table 3).

Germination at the elevated temperature of 18 °C resulted in visibly faster germination of the grains and associated root formation. The resulting green malt exhibited a characteristic cucumber-like odor and a slightly musty smell and appeared grayer than the malts produced at the previous temperatures. The increase in temperature during steeping and germination enhanced fungal growth and a rapid rise in toxin concentrations during malting. The concentrations of 3-AcDON, 15-AcDON, DON, and DON-3G are shown in Fig. 2d, and concentrations are listed in Table 3. In the green malt, DON levels reached 751–1952 µg/kg, representing 2981–7751% of the initial concentration in the raw barley. DON-3G showed an even more significant increase of up to 25,266% during germination, resulting in 1094–2458 µg/kg in the green malt, indicating a strong boost of enzymatic DON glycosylation at increased temperature during the malting process. After subsequent kilning, the malt with rootlets contained 1502–2587 µg/kg DON and 2375–3136 µg/kg DON-3G. Germ separation, including removing the highly contaminated rootlets, resulted in only minor changes to the toxin levels in the final kilned malt, with 1464–2421 µg/kg DON and 2323–3352 µg/kg DON-3G being detected. This corresponded to an average increase of 7086% for DON and 28,113% for DON-3G compared to the raw barley. The DON/DON-3G ratio during malting was 0.7 higher than in the comparable malting process at 14 °C. As in the previously conducted maltings, the acetylated DON derivatives were first detected and quantified during germination, starting from the green malt samples. In the green malt, 3-AcDON was found at 64.3–252 µg/kg concentrations and 15-AcDON at 4.68–8.96 µg/kg. The concentrations of these toxins continued to increase during kilning, resulting in final kilned malt levels of 209–421 µg/kg for 3-AcDON and 11.9–24.9 µg/kg for 15-AcDON (after germ separation), with 3-AcDON levels being significantly higher (at least tenfold) than its constitutional isomer. As with DON and DON-3G, removing the rootlets did not significantly reduce the toxin levels of the acetylated compounds.

#### Increase in spore concentration

An additional targeted inoculation with *F. culmorum* was performed using four times the standard spore concentration (4 × 10^5^CFU/mL) to examine the impact of the influence of the initial *F. culmorum* contamination. No visible infection was observed during the malting process in this case either. As in the previously described maltings, surface decontamination led to an average reduction in DON content by 56%, while the concentration of DON-3G changed only slightly. As with the artificial inoculation with a lower spore concentration, no significant changes in DON and DON-3G toxin levels were observed after 5 days of *F. culmorum* incubation. However, qPCR analysis indicated significant fungal growth independent of initial spore concentrations, suggesting that the fungus produced few toxins during the initial colonization of grains.

During germination, toxin levels increased significantly throughout the malting process. The DON content in the green malt samples reached 1706–3655% of the initial DON concentration in the raw barley. After kilning, a further increase in toxin concentration was noted, with the final kilned malt, after germ separation, containing 714–1571 µg/kg DON. This represented a 2733–6140% increase compared to the initial level. The highest DON concentrations, up to 5249 µg/kg, were quantified in the rootlets.

The modified toxin DON-3G rose sharply during germination, reaching levels up to 2824 µg/kg in the green malt, and continued to increase after kilning. From the green malt stage onward, the levels of DON-3G significantly exceeded those of the aglycone. After germ separation, the final kilned malt contained 2009–3856 µg/kg DON-3G, averaging 29,800% of the initial barley concentration. Similar to DON, the highest concentrations of DON-3G were found in the removed rootlets with values up to 7585 µg/kg. 3-AcDON and 15-AcDON were only detected in the samples after the completion of germination. These concentrations continued to increase during kilning, resulting in final kilned malt after germ separation containing 180–358 µg/kg 3-AcDON and 18.2–29.4 µg/kg 15-AcDON. The 3-AcDON content exceeded that of 15-AcDON by a factor of 10 in the green malt, malt, and rootlet samples. The type B trichothecene concentrations are listed in Table 3 and visualized in Fig. 2e.

### Analysis of fungal DNA

#### General remarks

The *F. culmorum* (*Fc*) DNA content normalized to barley DNA content was determined using qPCR in the samples taken during the various malting processes. This verified the success of the targeted artificial inoculation of the barley grains and enabled tracking of the progression of fungal DNA during malting. The DNA contents are listed in Table 3 as well as in the supplementary material (Table S2–S6). In a subsequent step, the obtained DNA data were correlated with the toxin data for DON, DON-3G, and potentially also for 3-AcDON and 15-AcDON.

#### Raw material

The initial raw barley material already exhibited low levels of *Fc* DNA concentration, with 0.0013 pg *Fc* DNA/ng barley DNA, indicating very little natural infection by *F. culmorum*. The surface disinfection of the samples and a 5-day incubation without prior artificial inoculation with fungal spores (“control”) did not significantly change the average DNA content. In the final malt after germ separation, an average of 0.0007 pg *Fc* DNA/ng barley DNA was found for the control malting, which is less than in the raw grain. The highest levels were detected in the removed rootlets, with an average content of 0.003 pg *Fc* DNA/ng barley DNA.

#### Standard malting procedure

Targeted artificial inoculation with *F. culmorum* (spore concentration, 10^5^ CFU/mL) showed a two- to threefold increase in fungal DNA after 5 days of incubation of the barley samples at 25 °C and 96% RH, thus confirming the success of the inoculation and the growth of *F. culmorum*. The DNA content in the green malt, averaging 0.19 pg *Fc* DNA/ng barley DNA, showed a further increase during germination. The removed rootlets contained the highest levels of fungal DNA, averaging 0.48 pg *Fc* DNA/ng barley DNA. In the malt after germ separation, an average of 0.21 pg *Fc* DNA/ng barley DNA was determined.

#### Variations of the malting process

The analysis of varying malting temperatures showed differing impacts on fungal growth. Lowering the malting temperature by 4 to 10 °C resulted in reduced fungal growth and, therefore, lower fungal DNA content, which was approximately three times lower in the green malt, averaging 0.06 pg *Fc* DNA/ng barley DNA, compared to malting at 14 °C. The standardized kilning conditions also led to further fungal growth in this malting approach, with average DNA contents of 0.08 pg *Fc* DNA/ng barley DNA in malt with rootlets. After germ separation, 0.06 pg *Fc* DNA/ng barley DNA was found in the cleaned malt, which is 70% less than in malting at 14 °C. Increasing the germination temperature to 18 °C resulted in much higher levels of *Fc* DNA in the green malt samples, averaging 16.4 pg *Fc* DNA/ng barley DNA. Post-kilning, an average of 7.54 pg *Fc* DNA/ng barley DNA remained in the finished brewing malt after germ separation. This represented a 3687% increase compared to malting at 14 °C. The rootlets from the malting samples at 18 °C contained the most fungal DNA with an average of 113 pg *Fc* DNA/ng barley DNA. Inoculation with four times the spore concentration of *F. culmorum* (4 × 10^5^ CFU/mL) resulted in an increase in fungal DNA shortly before and during the malting process. Starting from the initial contamination of the samples with 0.0013 pg *Fc* DNA/ng barley DNA, an average of 44.4 pg *Fc* DNA/ng barley DNA was determined after the incubation phase. Following germination, no significant differences in fungal DNA content were observed in the green malt, with an average of 43.6 pg *Fc* DNA/ng barley DNA across all biological replicates. In the malt after germ separation, an average of 35.3 pg *Fc* DNA/ng barley DNA was quantified, while the rootlets contained 5.10 pg *Fc* DNA/ng barley DNA. Thus, a more than 17,000% increase in fungal DNA content was determined in the malt due to the fourfold spore concentration.

#### Correlation of fungal DNA and toxin contents over the malting processes

To associate the observed variations in *F. culmorum* DNA concentrations with the mycotoxin levels throughout the malting processes, Pearson’s correlations were calculated, excluding the rootlets samples from the calculations. The correlations are presented in Table 4. The Pearson correlation coefficients generally show positive correlations between the *F. culmorum* DNA and the toxin levels. An exception is the malting at reduced germination temperature (10 °C), where the correlation between DON and fungal DNA was negative. For a spore concentration of 10^5^ CFU/mL *F. culmorum* and germination temperatures of 14 °C and 18 °C, significant two-tailed correlations were observed at a significance level of 0.01 between the fungal DNA content and the concentrations of 3-AcDON, 15-AcDON, DON, and DON-3G. In contrast, the correlation could not be significantly demonstrated (*p* > 0.05) with a fourfold spore concentration. Additionally, inconsistent correlations were noted between the DNA and toxin data in the malting at 10 °C, where generally very low DNA levels were determined in the process samples.

**Table 4:** Pearson’s correlation coefficients between *F. culmorum* DNA and the toxin concentrations of DON, DON-3G, 3-AcDON, and 15-AcDON during the different malting processes summarized across all process samples

Malting temperature	Spore amount	Mycotoxin			
		DON	DON-3G	3-AcDON	15-AcDON
10 °C	10^5^ CFU/mL	− 0.40	0.26	–	–
14 °C	10^5^ CFU/mL	0.91**	0.98**	0.94**	0.85**
18 °C	10^5^ CFU/mL	0.71**	0.74**	0,66**	0.58**
14 °C	4 × 10^5^ CFU/mL	0.41	0.46*	0.46	0.41
14 °C	4 × 10^5^ CFU/mL	0.71**^/+^	0.79**^/+^	0.78**^/+^	0.70**^/+^

^**^Correlation is statistically significant at *p* $\le$ 0.01 (two-tailed); *correlation is statistically significant at *p* $\le$ 0.05 (two-tailed); **^/+^correlation is statistically significant at *p* $\le$ 0.01 (two-tailed, if samples “5 days incubated” are not included)

The qPCR revealed particularly low DNA concentrations for the control malting and malting at a reduced temperature (10 °C). These were considered as practically *F. culmorum* free at DNA levels below 0.001 pg *Fc* DNA/ng barley DNA (Linkmeyer et al. 2013).

### Discussion of the malting processes

#### Influence of surface disinfection

The initial contamination of barley with BEA, DON, DON-3G, ENNs, and HT-2 indicated low prior contamination with toxin-producing *Fusarium* species in the field. FUSX, NIV, and T-2 were undetectable during the malting process, as also reported by Lancova et al. (2008). The surface disinfection of the grains, which involved submerging the grains in an H_2_O_2_ solution and sterile tap water for several minutes, resulted in the washing out of DON and, consequently, a reduction in its concentration. However, this effect was not observed for DON-3G, which can be predominantly found in vacuoles (Coleman et al. 1997). Habler et al. (2016c) also showed that the DON-3G content remained unchanged during steeping. Thus, the DON/DON-3G ratio, predominantly > 1 in the raw grain, changed to an average of 0.95 after surface treatment. A comparison of the toxin levels of DON and DON-3G after surface disinfection across the two conducted malting processes (targeted and control) showed nearly identical levels between the two processes.

#### Inoculation with *F. culmorum*

Inoculation with *F. culmorum* spores showed no increase in DON or DON-3G concentrations after incubation. However, qPCR analysis confirmed fungal growth during the 5 days through increased fungal DNA. Soaking of the grains has been shown by Schwarz et al. (1995), Habler et al. (2016c), and Maul et al. (2012) to wash out polar toxins like DON, which are predominantly located in the outer layers of the barley, into the soaking water. This was particularly true for DON. A similar phenomenon was observed during the surface disinfection earlier in this study, and this phenomenon has been repeatedly demonstrated in the literature, so an analysis of the soaking water was omitted. Germination and the associated water uptake by the grain enabled fungal growth and promoted mycotoxin production. The significant increase in DON-3G content from the green malt samples can be explained by the increased availability of glucose from starch metabolism and the heightened enzymatic activity, particularly of UDP-glucosyltransferases, in the barley grains during germination (Schweiger et al. 2010). Pascari et al. (2019), Vaclavikova et al. (2013), and Habler et al. (2016c) also observed a rapid increase of this modified toxin during germination and kilning in artificially field-infected barley samples. The results for DON-3G obtained here are thus in line with the literature. DON and 3-AcDON levels continued to rise after steeping, with subsequent kilning having only a marginal effect on toxin concentrations. The latter can be explained by the thermostability of the toxins, including DON-3G, up to 120 °C (Schwarz et al. 1995; Lancova et al. 2008; Vegi et al. 2011; Pascari et al. 2019). The comparison of toxin contents calculated per dry weight for “green malt” and “malt” reveals not only their temperature stability but also the ongoing formation of toxins, notably during the withering phase due to active UDP-glucosyltransferase, as observed by Habler et al. (2016c). This was also seen in most of the malting variations discussed later. Lancova et al. (2008) and Habler et al. (2016c) reported increases in DON and 3-AcDON levels during malting of field-infected samples of up to 220% for DON and 620% for 3-AcDON. The findings for DON in our study were thus consistent with those in the literature. No 3-AcDON was detected in the starting barley and only became quantifiable from the green malt samples onwards. The 3-AcDON content, as in the literature, was ≤ 10% of the DON content in the final kilned malt and was thus comparable (Kostelanska et al. 2009; Habler et al. 2016c). The significantly greater increase in DON-3G content compared to its aglycone during malting also resulted in a change in the toxin ratio. The DON/DON-3G ratio in the starting barley was 2.6, which decreased to 1.1 after surface disinfection and further to 0.3 from the green malt stage onwards. Higher DON-3G levels in malt compared to its aglycone were also observed in studies of various malt samples by Kostelanska et al. (2009), Kostelanska et al. (2011b), and Habler and Rychlik (2016). In these studies, DON/DON-3G ratios of < 1 were determined in malt, which could be explained by the higher glycosylation rate of the plant than the production rate of DON by the fungus. Additionally, hydrolytic enzymes can release DON-3G from the matrix during germination (Maul et al. 2012; Pascari et al. 2022). In the cleaning step, the machine removed the rootlets, some outer parts, and other byproducts. However, the fact that only marginally lower toxin concentrations of type B trichothecenes were detected in malt after germ separation compared to malt with rootlets shows that a reduction in toxin concentration by merely removing the rootlets is insufficient. Therefore, toxins are not limited to the rootlets or outer husks of the barley kernel. The removed parts were highly contaminated with toxins, yet due to the low weight of the rootlets, their removal did not alter the overall content of the final malt much. However, as the byproduct, including the rootlets, is often used as animal feed in agriculture, the high contamination with mycotoxins should not be overlooked (Zachariasova et al. 2014; Neylon et al. 2020). Significant differences in the biological triplicates were observed at the beginning of malting. The differences in toxin levels between biological replicates could be attributed to the uneven field contamination of the raw material or to technical variations of the artificial inoculation with spore suspension before malting. However, the DON-to-DON-3G ratio between biological replicates remained consistent despite high concentration differences, confirming the assumptions made in our study.

#### Control sample

The incubation at 25 °C and 96% RH following surface disinfection showed no significant difference in the toxin levels of DON and DON-3G in the control malting process, and also, the DON/DON-3G ratio remained unchanged. Thus, favorable conditions for fungal growth over several days had no impact on the toxin concentrations of DON and DON-3G. During the subsequent malting process, including the two steeping phases, the concentration of DON further decreased, as was already described by Habler et al. (2016c) and Lancova et al. (2008), either passing into the steeping water or being metabolized. Consequently, in the control malting process, DON could only be detected below the LOQ in green malt and malt, which is consistent with the determined *Fc* DNA levels. Therefore, further assessment of the DON/DON-3G ratio in the control malting process was impossible for subsequent samples.

#### Influence of malting parameters

To investigate the effects of varying spore concentrations and malting temperatures on toxin formation, further experiments were conducted with barley samples inoculated with higher spore loads or malted at 10 °C and 18 °C, respectively. Comparative experimental setups with a defined targeted inoculation at the beginning have not been published yet, so a direct comparison with the literature was not possible. However, the effects of malting parameters on the growth of microorganisms have been partially studied, allowing conclusions to be drawn about the associated potential of mycotoxin formation (van Nierop et al. 2006; Laitila et al. 2007; Prusova et al. 2022).

#### Temperature

Lowering the germination temperature to 10 °C resulted in significantly reduced fungal DNA levels, which were mainly below the LOD. This consequently led to lower toxin concentrations in the malt. The DON/DON-3G ratio changed during the experiment from 2.6 in the raw barley to 0.4 in the green malt samples and 1.4 in the final kilned malt (after cleaning). The reduced overall production of the studied mycotoxins and the minimal detection of *Fusarium* DNA in the samples demonstrated that 10 °C was not optimal for the growth of *F. culmorum*. This was to be expected, as the optimal growth of this fungus was reported to be in a range of 20–25 °C (Brennan et al. 2003). Cooler temperatures also significantly slowed and reduced the de novo production of DON during malting. This reduction in DON synthesis consequently decreased the plant’s detoxification response, which involves glycosylating DON into DON-3G. The 16-h withering at 50 °C after germination caused a noticeable increase in DON concentration by the fungus without instant plant response, explaining the altered ratio of the aglycone to its modified form at 1.4. Frandsen et al. (2000) demonstrated that the highest enzymatic activity of glycosyltransferase occurs 48–72 h after the start of the germination process, which is earlier than the increase of DON concentration during withering and helps to explain the quick change of the toxin ratios from 0.4 to 1.4. The increase in DON might also result from the release of higher-glycosylated DON-oligo-glucosides, as described by Zachariasova et al. (2012). In summary, lowering the germination temperature to 10 °C strongly influenced the growth of *F. culmorum* and the associated mycotoxin production during the malting process. This effect was particularly evident in the DON-3G levels at low temperatures, which were, on average, 5–6 times lower than at 14 °C. The DON/DON-3G ratio in the kilned malt was also notably higher than at the MEBAK standard temperature of 14 °C.

Germination at 18 °C resulted in higher contents of *Fc* DNA in the samples than at 10 °C and 14 °C. This went along with higher concentrations of the relevant mycotoxins 3-AcDON, 15-AcDON, DON, and DON-3G. The DON/DON-3G ratio, which was similar in the raw barley, post-surface disinfection, and after 5 days of incubation to the previous malting experiments, began to change with the malting process. Both the green malt and malt samples showed a DON/DON-3G concentration ratio of 0.7, nearly twice as high as in the comparative malting at 14 °C (ratio 0.3). Kokkonen et al. (2010) studied the effect of water activity and temperature on mycotoxin concentrations of various *Fusarium* species cultured on grain mixtures (wheat, oats, barley), basing their findings on storage experiments rather than on germination. They found, similar to the present study, that higher temperatures led to the production of significantly more DON and 3-AcDON compared to lower temperatures without relating their results to fungal biomass. Prusova et al. (2022) investigated the impact of a higher malting temperature (increasing from 15 to 20 °C) on type B trichothecenes NIV, neosolaniol, and type A compounds HT-2, T-2, HT-2-G, T-2-G and the depsipeptides ENNs and BEA during the malting and brewing process. They demonstrated that increasing the germination temperature by 5 °C resulted in an average tripling of *Fusarium* toxin concentrations. In the current study, concentration increases by up to a factor of 10 were recorded with a 4 °C increase in malting temperature. However, this is mainly attributed to much higher fungal biomass than to higher toxin production per fungal unit.

#### Spore concentration

An increase in spore concentration was expected to lead to enhanced fungal growth and, consequently, higher toxin production. A fourfold increase in spore concentration resulted in a significant rise in the toxin concentrations of 3-AcDON and DON produced by *F. culmorum* and also of the plant metabolite DON-3G, exceeding four times the initial levels. However, the DON-to-DON-3G ratio remained comparable to the previous malting experiments, suggesting that the enzyme activities in the barley kernels were similar due to consistent malting parameters such as temperature and relative humidity. Thus, only the spore count/mycelium size varied. The increased production of DON subsequently led to a greater conversion to DON-3G. The higher spore count allowed more extensive fungal growth on the grains, resulting in more toxin production; however, fungal growth did not increase linearly with the spore concentration as fungal DNA was not increased to the same extent. Mycotoxin production in fungi is generally a complex process that is not fully understood to this day. It is regulated by genetic mechanisms that respond to various environmental stimuli, such as temperature, nutrient availability and composition, or water activity, and further mycotoxin biosynthesis. The presence of other microorganisms and the response of a host plant can also impact fungal growth and activity (Bottalico & Perrone 2002; Kokkonen et al. 2010). 3-AcDON and 15-AcDON were detectable only starting from the green malt samples, as observed in the previous inoculation with lower spore concentration. However, these toxins were present in significantly higher concentrations in the experiment with higher spore concentrations.

#### Correlation of fungal DNA and toxin contents over the malting processes

Pearson’s correlations were calculated to associate the observed variations in *F. culmorum* DNA concentrations with the mycotoxin levels throughout the malting processes. With a spore concentration of 10^5^ CFU/mL, significant positive correlations were observed between the toxin levels and the fungal DNA concentrations (*p* < 0.01). The malt samples showed clear contamination with *F. culmorum* compared to the control malting, attributable to the targeted artificial inoculation. Habler et al. (2016c) also found significant correlations between *F. culmorum* DNA content and mycotoxin levels of 3-AcDON, 15-AcDON, DON, and DON-3G throughout the malting process in barley previously infected in the field. Moreover, Nielsen et al. (2014), Linkmeyer et al. (2013), and Hoheneder et al. (2022) demonstrated positive, primarily significant correlations between *F. culmorum* DNA content and DON and DON derivates in various barley varieties and samples in the field. However, they also pointed out that the association between fungal DNA content and corresponding mycotoxin concentrations depends not only on different barley cultivars but also on regional and seasonal influences and, thus, may not always be observed.

Lowering the barley germination temperature slowed fungal growth due to unfavorable conditions, resulting in lower mycotoxin production. The low DNA levels also explain why no significant correlations between fungal DNA levels and corresponding mycotoxins could be established in this experiment. Increasing the malting temperatures to 18 °C created conditions close to the optimal growth range for *F. culmorum* (20–25 °C), as indicated by a marked increase in fungal growth reflected in the DNA content. Despite this, no visually detectable mycelium formation occurred, even at elevated germination temperatures. This suggests that *F. culmorum* invaded germinating barley seedlings rather than growing on its surface. This would also explain the relatively high DON-3G contents throughout our study because this metabolite is likely formed when metabolically active plant cells rather than senescent barley hulls come into contact with DON and metabolize the toxin with the help of UDP-glycosyltransferases. The quadrupling of spore concentration increased contamination with *F. culmorum*. A positive correlation between DNA concentration and toxin levels was again observed, although this was mostly not significant (*p* > 0.05). Only for DON-3G significant correlations between fungal DNA content and toxin concentrations were observed at *p* ≤ 0.05 throughout the process. Since the surface of the barley grains used in the malting trials was initially decontaminated with H_2_O_2_, the initial microbial load of the barley was minimized. It is believed that some molds produce certain mycotoxins during their growth to protect against other competing fungi or bacteria competing for the same nutrients or to help colonize the host plant by weakening its defense mechanisms (Desmond et al. 2008; Walter et al. 2010; Kazan et al. 2012). While the fungus was growing on the grains after 5 days of incubation, as indicated by the high DNA levels, no toxin production occurred. Toxin synthesis began only with the start of wet steeping and the associated increase in the water content of the grains. If the samples with high fungal DNA but low DON content (“5 days incubated grain”) are excluded from the correlation calculations, significant two-tailed correlations at *p* < 0.01 with coefficients between 0.70 and 0.79 for DON, DON-3G, 3-AcDON, and 15-AcDON are observed even at quadrupled spore concentration (Table 4).

On an industrial scale, barley is often mixed during steeping using compressed air, which may help remove water-soluble toxins more effectively. Although this method was not technically feasible in our malting trials, oxygen introduction and constant circulation during steeping were ensured. To minimize stress conditions, such as elevated CO_2_ levels during malting, the germinated material in the germination containers was turned twice daily to provide aeration. However, increased toxin production as a stress response from the *F. culmorum* strain, particularly under higher germination temperatures and the associated increased respiration of the kernels, cannot be ruled out and has been reported in the literature (Magan et al. 2011; Bencze et al. 2017).

To conclude, in our study, strong changes in the relative and absolute toxin amounts occurred during the malting process. These effects were very pronounced in our study under defined conditions and allowed us to deduce patterns in toxin formation and distribution during the malting process. These patterns (i.e., the increase of DON-3G during malting) will also be seen in scenarios without prior targeted inoculation if natural infection is high due to seasonal particularities. However, this may be less reproducible and little predictable, but it likely impacts the malting and brewing processes. This emphasizes the relevance of analyzing mycotoxins in malting barley before usage in breweries or measuring fungal DNA or toxins in raw barley to eliminate potentially risky batches before they enter the malting process.

We further showed that technological means to influence mold growth and toxin formation during the malting process are limited. The influence of malting temperature on mold growth and the associated mycotoxin production was evident in the malting trials conducted. Lowering the malting temperature from 14 to 10 °C significantly reduced fungal growth and toxin concentrations. However, the lower temperature also decreased germination capacity, which could lead to poorer malt quality. Therefore, the malting temperature of 14 °C recommended by MEBAK can be considered a good compromise between minimizing toxin production and maintaining kernel germination capacity. A potential reduction in toxins in the malting process could be achieved through more intensive steeping and shorter germination phases. Both aspects were not investigated in this study but can be deducted from our results. More intense steeping will lead to increased “wash-out” of toxins, and a shorter germination phase might result in less fungal biomass, thus mitigating mycotoxin production. However, a shortened germination time also results in less pronounced starch mobilization, which is essential for the subsequent brewing process.

Thus, the choice of raw material is one of the few parameters that affect the toxin content in the final malt. The initial microbial contamination of barley is crucial for the potential growth of microorganisms, especially fungi, during the malting process. Our study clearly showed that initial toxin contamination in the raw barley is transferred more or less to the final malt, with only minor amounts being “washed out” during steeping. The choice of more resistant barley varieties can significantly affect toxin contamination levels already in the field (Hoheneder et al. 2022), leading to lower toxin levels during malting and thus contributing to better malt quality.

The high levels of DON-3G, alongside DON and its acetylated derivatives, highlight the potential need for regulatory frameworks to encompass these modified toxins because they may similarly occur in samples with naturally high infection levels, as already shown by Jin et al. (2018) and Ksieniewicz-Woźniak et al. (2019). Current regulations that focus primarily on DON overlook the potential health risks posed by its derivatives, such as DON-3G, especially considering their concentration increase during the malting process. The findings of this study suggest that existing maximum limits may only fully protect consumers if they account for the total burden of DON-related toxins. Thus, authorities should reconsider and update mycotoxin regulations to include these modified forms, ensuring comprehensive monitoring and control throughout the entire food production chain.

#### Outlook

The developed malting system can be utilized in the future to investigate additional *Fusarium* species and other barley cultivars. For barley types resistant in the field, it would be pertinent to determine whether they maintain this resistance during malting or if they produce particularly high levels of DON-3Glc, as this could be a resistance mechanism of the plant (Bethke et al. 2023). Artificial inoculation offers a significant advantage here, allowing researchers to overcome the noise of often low natural infection rates, thereby enabling the detection of effects that would otherwise be unmeasurable. This approach holds great potential for further research.

## Supplementary Information

Below is the link to the electronic supplementary material.

Supplementary file1 (DOCX 62.1 KB)

## Data Availability

No datasets were generated or analysed during the current study.

## Abbreviations

15-AcDON: 15-Acetyldeoxynivalenol
3-AcDON: 3-Acetyldeoxynivalenol
BEA: Beauvericin
CFU: Colony forming unit
DON: Deoxynivalenol
DON-3G: Deoxynivaenol-3-glucoside
ENN: Enniatin
ESI: Electrospray ionization
FUSX: Fusarenone X
HT-2: HT-2 toxin
LC–MS/MS: Liquid chromatography-tandem mass spectrometry
MEBAK: Mitteleuropäische Brautechnische Analysenkommission e.V.
NIV: Nivalenol
qPCR: Quantitative polymerase chain reaction
RH: Relative humidity
RSD: Relative standard deviation
SD: Standard deviation
SIDA: Stable isotope dilution assay
T-2: T-2 toxin
ZEN: Zearalenone
