# Supplementary material for: Monitoring Fusarium toxins from barley to malt: Targeted inoculation with Fusarium culmorum
Source: Mycotoxin Res. 2024 Dec 20;41(1):215–37. doi: 10.1007/s12550-024-00573-y (PMC11757896; doi:10.1007/s12550-024-00573-y)
Supplement: Supplementary file 1 — Supplementary file1 (DOCX 62.1 KB) [file 12550_2024_573_MOESM1_ESM.docx]

**Table S1:** Spiking level used for the determination of LOD, LOQ, precisions, and recovery of each *Fusarium* toxin.

|  | LOD and LOQ in µg/kg | | | |  | Precision in µg/kg |  | Recovery in µg/kg | | |
| --- | --- | --- | --- | --- | --- | --- | --- | --- | --- | --- |
|  | Level 1 | Level 2 | Level 3 | Level 4 |  | Level 1 |  | Level 1 | Level 2 | Level 3 |
| NIV | 1.5 | 4.5 | 7.5 | 15 |  | 15 |  | 7.5 | 15 | 50 |
| DON | 0.75 | 2 | 5 | 7.5 |  | 50 |  | 50 | 300 | 500 |
| DON-3G | 2 | 5 | 10 | 20 |  | 100 |  | 50 | 100 | 300 |
| ZEN | 0.15 | 0.3 | 0.75 | 1.5 |  | 20 |  | 1.5 | 10 | 20 |
| FUSX | 3 | 7.5 | 15 | 30 |  | 50 |  | 15 | 30 | 50 |
| 3-AcDON | 0.5 | 1 | 2.5 | 5 |  | 10 |  | 10 | 20 | 70 |
| 15-AcDON | 2 | 5 | 10 | 20 |  | 30 |  | 30 | 70 | 100 |
| HT-2 | 0.05 | 0.1 | 0.25 | 0.5 |  | 5 |  | 5 | 10 | 50 |
| T-2 | 0.02 | 0.05 | 0.1 | 0.2 |  | 2 |  | 2 | 5 | 10 |
| ENN A | 0.02 | 0.05 | 0.1 | 0.2 |  | 7 |  | 7 | 15 | 50 |
| ENN A1 | 0.03 | 0.08 | 0.15 | 0.3 |  | 7 |  | 7 | 15 | 50 |
| ENN B | 0.01 | 0.03 | 0.05 | 0.1 |  | 7 |  | 7 | 15 | 50 |
| ENN B1 | 0.01 | 0.03 | 0.05 | 0.1 |  | 7 |  | 7 | 15 | 50 |
| BEA | 0.02 | 0.05 | 0.1 | 0.2 |  | 7 |  | 1 | 7 | 15 |

**Table S2**: Detailed mycotoxin concentrations from inoculation with F. culmorum (10^5^ CFU/mL) at 14 °C across three biological replicates. Data are presented in µg/kg ± SD. All values are given as the mean of triplicate determinations and duplicate injections. Toxins not listed in the table were not detected in any sample. B = barley, M = malt. n. d. = not detected, LOQ = limit of quantification.

|  | **Sample type** | ***Fc* DNA** | **DON** | **DON-3G** | **3-AcDON** | **15-AcDON** | **HT-2** | **ENN A** | **ENN A1** | **ENN B** | **ENN B1** | **BEA** |
| --- | --- | --- | --- | --- | --- | --- | --- | --- | --- | --- | --- | --- |
|  |  | **pg/ng B. DNA** | **[µg/kg]** | **[µg/kg]** | **[µg/kg]** | **[µg/kg]** | **[µg/kg]** | **[µg/kg]** | **[µg/kg]** | **[µg/kg]** | **[µg/kg]** | **[µg/kg]** |
| Replicate 1 | B. raw | 0.0013 | 25.2 ± 2.98 | 9.69 ± 0.35 | *n. d.* | *n. d.* | 3.84 ± 0.16 | 0.08 ± 0.01 | 0.43 ± 0.01 | 3.51 ± 0.14 | 1.35 ± 0.05 | 1.83 ± 0.12 |
|  | B. surface disinfected | 0.0006 | 13.1 ± 0.25 | 13.1 ± 1.23 | *n. d.* | *n. d.* | 1.47 ± 0.02 | *< LOQ* | 0.29 ± 0.01 | 13.2 ± 1.02 | 10.5 ± 0.41 | 0.38 ± 0.03 |
|  | B. 5 days incubated | 0.0059 | 14.2 ± 0.38 | 11.0 ± 0.28 | n. d. | *n. d.* | 3.48 ± 0.03 | *n. d.* | *< LOQ* | 2.81 ± 0.09 | 2.48 ± 0.04 | 1.58 ± 0.08 |
|  | Green malt | 0.1400 | 21.8 ± 0.89 | 79.0 ± 11.7 | 4.13 ± 0.18 | *< LOQ* | 1.75 ± 0.03 | *n. d.* | 0.86 ± 0.02 | 53.7 ± 6.43 | 38.5 ± 1.58 | 0.53 ± 0.02 |
|  | M. with rootlets | 0.2482 | 30.4 ± 0.88 | 213 ± 21.6 | 3.79 ± 0.30 | 3.81 ± 0.10 | 2.10 ± 0.07 | *< LOQ* | *< LOQ* | 4.33 ± 0.08 | 3.26 ± 0.16 | 1.09 ± 0.10 |
|  | M. after germ separation | 0.0923 | 26.5 ± 2.48 | 72.4 ± 4.57 | 3.57 ± 0.15 | 2.90 ± 0.11 | 3.42 ± 0.01 | *< LOQ* | 0.64 ± 0.02 | 51.2 ± 3.80 | 20.2 ± 0.77 | 1.07 ± 0.06 |
|  | Rootlets | 0.6730 | 189 ± 4.43 | 241 ± 5.27 | 18.7 ± 0.33 | 3.72 ± 0.25 | 34.4 ± 0.58 | *n. d.* | 2.58 ± 0.08 | 55.0 ± 2.51 | 29.0 ± 1.98 | 6.90 ± 0.61 |
| Replicate 2 | B. raw | 0.0013 | 25.2 ± 2.98 | 9.69 ± 0.35 | *n. d.* | *n. d.* | 3.84 ± 0.16 | 0.08 ± 0.01 | 0.43 ± 0.01 | 3.51 ± 0.14 | 1.35 ± 0.05 | 1.83 ± 0.12 |
|  | B. surface disinfected | 0.0009 | 19.8 ± 1.20 | 16.8 ± 1.42 | *n. d.* | *n. d.* | 3.43 ± 0.11 | *< LOQ* | 1.55 ± 0.03 | 97.8 ± 6.84 | 97.3 ± 3.17 | 0.34 ± 0.02 |
|  | B. 5 days incubated | 0.0287 | 15.6 ± 0.38 | 11.1 ± 0.55 | *n. d.* | *n. d.* | 3.45 ± 0.09 | *n. d.* | *< LOQ* | 7.88 ± 0.65 | 7.94 ± 0.46 | 0.19 ± 0.01 |
|  | Green malt | 0.1227 | 6.57 ± 0.43 | 30.2 ± 1.90 | 1.21 ± 0.05 | *< LOQ* | 1.23 ± 0.04 | *n. d.* | 8.50 ± 0.13 | 335 ± 19.4 | 259 ± 1.43 | 1.31 ± 0.09 |
|  | M. with rootlets | 0.1810 | 21.5 ± 1.48 | 61.8 ± 4.47 | 3.41 ± 0.27 | 3.02 ± 0.19 | 3.04 ± 0.01 | 0.19 ± 0.01 | 4.55 + 0.18 | 87.0 ± 2.85 | 44.2 ± 0.91 | 1.23 ± 0.07 |
|  | M. after germ separation | 0.0503 | 28.0 ± 2.60 | 80.1 ± 7.70 | 2.97 ± 0.14 | 3.03 ± 0.08 | 3.05 ± 0.02 | *< LOQ* | 1.76 ± 0.02 | 54.0 ± 2.09 | 38.0 ± 2.17 | 1.22 ± 0.02 |
|  | Rootlets | 0.1859 | 274 ± 18.0 | 332 ± 9.88 | 18.4 ± 0.17 | *< LOQ* | 77.6 ± 0.59 | *n. d.* | 3.90 ± 0.10 | 60.1 ± 0.97 | 48.8 ± 3.24 | 8.71 ± 0.40 |
| Replicate 3 | B. raw | 0.0013 | 25.2 ± 2.98 | 9.69 ± 0.35 | *n. d.* | *n. d.* | 3.84 ± 0.16 | 0.08 ± 0.01 | 0.43 ± 0.01 | 3.51 ± 0.14 | 1.35 ± 0.05 | 1.83 ± 0.12 |
|  | B. surface disinfected | 0.0029 | 17.1 ± 0.98 | 16.6 ± 1.32 | *n. d.* | *n. d.* | 3.48 ± 0.20 | *< LOQ* | 0.25 ± 0.01 | 7.30 ± 0.23 | 7.20 ± 1.43 | 0.47 ± 0.05 |
|  | B. 5 days incubated | 0.0751 | 17.1 ± 0.21 | 10.8 ± 1.20 | *n. d.* | *n. d.* | 5.02 ± 0.05 | 0.47 ± 0.02 | 13.5 ± 0.54 | 664 ± 21.2 | 112 ± 14.0 | 0.98 ± 0.03 |
|  | Green malt | 0.2943 | 113 ± 3.04 | 219 ± 31.3 | 17.5 ± 1.03 | *< LOQ* | 3.27 ± 0.08 | *n. d.* | *< LOQ* | 3.34 ± 0.30 | 5.36 ± 0.45 | 1.03 ± 0.02 |
|  | M. with rootlets | 0.6181 | 176 ± 11.9 | 476 ± 44.8 | 24.4 ± 2.78 | 5.03 ± 0.32 | 27.8 ± 0.56 | 0.18 ± 0.01 | 2.93 ± 0.03 | 14.6 ± 0.88 | 10.7 ± 0.37 | 1.93 ± 0.10 |
|  | M. after germ separation | 0.4713 | 153 ± 16.5 | 373 ± 27.9 | 25.9 ± 1.33 | 7.11 ± 0.28 | 3.96 ± 0.26 | *< LOQ* | 1.74 ± 0.03 | 41.8 ± 1.64 | 34.6 ± 1.80 | 1.61 ± 0.04 |
|  | Rootlets | 0.5831 | 414 ± 27.7 | 609 ± 57.9 | 51.0 ± 3.78 | 9.50 ± 0.27 | 52.0 ± 0.08 | *n. d.* | 0.65 ± 0.04 | 26.4 ± 4.64 | 12.1 ± 0.78 | 8.01 ± 0.20 |

**Table S3:** Detailed mycotoxin concentrations from control malting trial (inoculation with sterile tap water) at 14 °C across three biological replicates. Data are presented in µg/kg ± SD. All values are given as the mean of triplicate determinations and duplicate injections. Toxins not listed in the table were not detected in any sample. B = barley, M = malt. n. d. = not detected, LOQ = limit of quantification.

|  | **Sample type** | **Fc DNA** | **DON** | **DON-3G** | **3-AcDON** | **HT-2** | **ENN A** | **ENN A1** | **ENN B** | **ENN B1** | **BEA** |  |
| --- | --- | --- | --- | --- | --- | --- | --- | --- | --- | --- | --- | --- |
|  |  | **pg/ng B. DNA** | **[µg/kg]** | **[µg/kg]** | **[µg/kg]** | **[µg/kg]** | **[µg/kg]** | **[µg/kg]** | **[µg/kg]** | **[µg/kg]** | **[µg/kg]** |  |
| Replicate 1 | B. raw | 0.0013 | 25.2 ± 2.98 | 9.69 ± 0.35 | *n. d.* | 3.84 ± 0.16 | 0.08 ± 0.01 | 0.43 ± 0.01 | 3.51 ± 0.14 | 1.35 ± 0.05 | 1.83 ± 0.12 |  |
|  | B. surface disinfected | 0.0052 | 12.3 ± 0.15 | 13.7 ± 0.26 | *n. d.* | 1.63 ± 0.03 | 0.20 ± 0.02 | 1.94 ± 0.09 | 8.46 ± 0.75 | 5.23 ± 0.44 | 5.48 ± 0.37 |  |
|  | B. 5 days incubated | 0.0018 | 11.2 ± 0.43 | 13.2 ± 0.84 | *n. d.* | 2.12 ± 0.16 | *< LOQ* | 0.33 ± 0.01 | 3.25 ± 0.12 | 1.25 ± 0.08 | 0.41 ± 0.03 |  |
|  | Green malt | 0.0101 | *< LOQ* | 6.34 ± 0.22 | *n. d.* | 0.45 ± 0.01 | *< LOQ* | *< LOQ* | 2.15 ± 0.07 | 0.95 ± 0.08 | 0.44 ± 0.03 |  |
|  | M. with rootlets | 0.0002 | *< LOQ* | 16.9 ± 1.04 | *n. d.* | 1.29 ± 0.07 | *n. d.* | 1.50 ± 0.11 | 19.6 ± 0.71 | 8.52 ± 0.48 | 1.15 ± 0.14 |  |
|  | M. after germ separation | 0.0011 | *< LOQ* | 10.9 ± 0.48 | *n. d.* | 1.75 ± 0.01 | *n. d.* | 1.21 ± 0.18 | 11.0 ± 0.53 | 6.05 ± 0.78 | 0.83 ± 0.03 |  |
|  | Rootlets | 0.0024 | *n. d.* | 87.3 ± 4.20 | *< LOQ* | 3.54 ± 0.11 | *n. d.* | 2.72 ± 0.18 | 41.7 ± 0.50 | 17.8 ± 0.30 | 4.53 ± 0.45 |  |
| Replicate 2 | B. raw | 0.0013 | 25.2 ± 2.98 | 9.69 ± 0.35 | *n. d.* | 3.84 ± 0.16 | 0.08 ± 0.01 | 0.43 ± 0.01 | 3.51 ± 0.14 | 1.35 ± 0.05 | 1.83 ± 0.12 |  |
|  | B. surface disinfected | 0.0003 | 12.4 ± 0.37 | 13.4 ± 0.34 | *n. d.* | 2.61 ± 0.16 | *n. d.* | 0.35 ± 0.03 | 5.56 ± 0.59 | 2.80 ± 0.05 | 0.20 ± 0.01 |  |
|  | B. 5 days incubated | 0.0009 | 13.2 ± 0.16 | 13.6 ± 0.39 | *n. d.* | 2.74 ± 0.02 | *n. d.* | 0.37 ± 0.01 | 5.77 ± 0.45 | 3.57 ± 0.22 | 0.17 ± 0.01 |  |
|  | Green malt | 0.0002 | *< LOQ* | 6.48 ± 0.18 | *n. d.* | 3.82 ± 0.35 | *n. d.* | *< LOQ* | 1.45 ± 0.11 | 0.51 ± 0.03 | 0.74 ± 0.11 |  |
|  | M. with rootlets | 0.0003 | *< LOQ* | 16.3 ± 0.20 | *n. d.* | 0.78 ± 0.01 | *n. d.* | 1.89 ± 0.31 | 123 ± 9.74 | 41.8 ± 1.08 | 1.09 ± 0.06 |  |
|  | M. after germ separation | 0.0011 | *< LOQ* | 9.85 ± 0.33 | *n. d.* | 0.96 ± 0.03 | *n. d.* | 1.96 ± 0.10 | 7.65 ± 0.43 | 5.25 ± 0.26 | 1.00 ± 0.09 |  |
|  | Rootlets | 0.0048 | *n. d.* | 42.5 ± 1.97 | *< LOQ* | 0.99 ± 0.04 | *n. d.* | 2.32 ± 0.39 | 9.64 ± 0.95 | 6.54 ± 0.34 | 5.26 ± 0.04 |  |
| Replicate 3 | B. raw | 0.0013 | 25.2 ± 2.98 | 9.69 ± 0.35 | *n. d.* | 3.84 ± 0.16 | 0.08 ± 0.01 | 0.43 ± 0.01 | 3.51 ± 0.14 | 1.35 ± 0.05 | 1.83 ± 0.12 |  |
|  | B. surface disinfected | 0.0029 | 13.0 ± 0.41 | 12.4 ± 0.59 | *n. d.* | 2.56 ± 0.08 | *n. d.* | 0.30 ± 0.05 | 5.60 ± 0.52 | 3.54 ± 0.27 | 0.67 ± 0.03 |  |
|  | B. 5 days incubated | 0.0011 | 15.6 ± 0.72 | 13.4 ± 0.54 | *n. d.* | 2.84 ± 0.03 | *n. d.* | 4.99 ± 0.84 | 300 ± 0.27 | 121 ± 1.25 | 0.67 ± 0.08 |  |
|  | Green malt | 0.0018 | *< LOQ* | 7.50 ± 0.16 | *n. d.* | 1.00 ± 0.04 | *n. d.* | *< LOQ* | 2.55 ± 0.23 | 0.95 ± 0.06 | 0.76 ± 0.10 |  |
|  | M. with rootlets | 0.0015 | *< LOQ* | 13.8 ± 0.19 | *n. d.* | 1.98 ± 0.02 | *n. d.* | *< LOQ* | 5.53 ± 0.42 | 2.01 ± 0.23 | 2.39 ± 0.14 |  |
|  | M. after germ separation | 0.0000 | *< LOQ* | 10.5 ± 0.46 | *n. d.* | 1.70 ± 0.09 | *n. d.* | 1.02 ± 0.09 | 30.4 ± 2.45 | 17.8 ± 1.31 | 2.47 ± 0.23 |  |
|  | Rootlets | 0.0018 | *n. d.* | 77.6 ± 1.93 | *< LOQ* | 2.50 ± 0.18 | *n. d.* | 0.81 ± 0.18 | 31.9 ± 2.49 | 10.6 ± 0.04 | 4.76 ± 0.24 |  |

**Table S4:** Detailed mycotoxin concentrations from inoculation with F. culmorum (10^5^ CFU/mL) at 10 °C across three replicates. Data are presented in µg/kg ± SD. All values are given as the mean of triplicate determinations and duplicate injections. Toxins not listed in the table were not detected in any sample. B = barley, M = malt. n. d. = not detected, LOQ = limit of quantification.

|  | **Sample type** | **Fc DNA** | **DON** | **DON-3G** | **3-AcDON** | **15-AcDON** | **HT-2** | **ENN A** | **ENN A1** | **ENN B** | **ENN B1** | **BEA** |
| --- | --- | --- | --- | --- | --- | --- | --- | --- | --- | --- | --- | --- |
|  |  | **pg/ng B. DNA** | **[µg/kg]** | **[µg/kg]** | **[µg/kg]** | **[µg/kg]** | **[µg/kg]** | **[µg/kg]** | **[µg/kg]** | **[µg/kg]** | **[µg/kg]** | **[µg/kg]** |
| Replicate 1 | B. raw | 0.0013 | 25.2 ± 2.98 | 9.69 ± 0.35 | *n. d.* | *n. d.* | 3.84 ± 0.16 | 0.08 ± 0.01 | 0.43 ± 0.01 | 3.51 ± 0.14 | 1.35 ± 0.05 | 1.83 ± 0.12 |
|  | B. surface disinfected | 0.0007 | 14.3 ± 0.18 | 8.94 ± 0.41 | *n. d.* | *n. d.* | 2.28 ± 0.06 | *< LOQ* | 0.40 ±0.05 | 6.06 ± 0.40 | 2.35 ± 0.19 | 0.47 ± 0.04 |
|  | B. 5 days incubated | 0.0037 | 15.1 ± 0.67 | 13,3 ± 0.44 | *n. d.* | *n. d.* | 9.19 ± 0.20 | *< LOQ* | 4.83 ± 0.34 | 162 ± 3.55 | 52.2 ± 13.2 | 0.46 ± 0.03 |
|  | Green malt | 0.0439 | 4.03 ± 0.13 | 7.93 ± 0.29 | *n. d.* | *n. d.* | 3.14 ± 0.11 | *< LOQ* | 0.40 ± 0.03 | 9.54 ± 0.65 | 2.93 ± 0.37 | 0.11 ± 0.01 |
|  | M. with rootlets | 0.1013 | 5.59 ± 0.31 | 13.8 ± 0.38 | *n. d.* | *n. d.* | 5.01 ± 0.47 | 0.12 ± 0.01 | 1.05 ± 0.14 | 12.4 ± 0.75 | 9.41 ± 1.87 | 1.03 ± 0.06 |
|  | M. after germ separation | 0.0544 | 20.2 ± 1.78 | 12.3 ± 0.60 | *n. d.* | *n. d.* | 1.99 ± 0.06 | 0.11 ± 0.01 | 1.84 ± 0.16 | 27.9 ± 2.13 | 8.47 ± 0.01 | 0.84 ± 0.04 |
|  | Rootlets | 0.1720 | 7.30 ± 0.64 | 97.7 ± 5.45 | 0.75 ± 0.07 | *< LOQ* | 4.62 ± 0.22 | 0.26 ± 0.00 | 1.32 ± 0.00 | 17.0 ± 0.94 | 5.89 ± 0.41 | 1.82 ± 0.21 |
| Replicate 2 | B. raw | 0.0013 | 25.2 ± 2.98 | 9.69 ± 0.35 | *n. d.* | *n. d.* | 3.84 ± 0.16 | 0.08 ± 0.01 | 0.43 ± 0.01 | 3.51 ± 0.14 | 1.35 ± 0.05 | 1.83 ± 0.12 |
|  | B. surface disinfected | 0.0000 | 15.8 ± 0.36 | 9.65 ± 0.22 | *n. d.* | *n. d.* | 1.82 ± 0.04 | *< LOQ* | 1.57 ± 0.32 | 13.5 ± 1.49 | 6.99 ± 1.03 | 0.46 ± 0.04 |
|  | B. 5 days incubated | 0.0070 | 21.4 ± 1.10 | 13.5 ± 0.23 | *n. d.* | *n. d.* | 2.11 ± 0.10 | *< LOQ* | 0.86 ± 0.04 | 41.0 ± 2.72 | 32.3 ± 3.80 | 0.89 ± 0.04 |
|  | Green malt | 0.0808 | 2.26 ± 0.09 | 6.00 ± 0.21 | *n. d.* | *n. d.* | 1.32 ± 0.08 | *< LOQ* | *< LOQ* | 8.24 ± 0.97 | 3.10 ± 0.34 | 3.69 ± 0.10 |
|  | M. with rootlets | 0.0700 | 28.8 ± 2.60 | 14.8 ± 0.98 | *n. d.* | *n. d.* | 2.31 ± 0.15 | 0.06 ± 0.00 | 0.54 ± 0.02 | 17.1 ± 0.91 | 16.7 ± 1.33 | 0.90 ± 0.08 |
|  | M. after germ separation | 0.0699 | 6.93 ± 0.46 | 13.7 ± 1.06 | *n. d.* | *n. d.* | 5.85 ± 0.25 | 0.11 ± 0.01 | 0.99 ±0.05 | 4.57 ± 0.35 | 4.51 ± 0.53 | 1.14 ± 0.03 |
|  | Rootlets | 0.3733 | 7.51 ± 0.43 | 95.1 ± 4.06 | 1.51 ± 0.15 | *< LOQ* | 3.10 ± 0.17 | 0.23 ± 0.01 | 0.58 ± 0.03 | 3.12 ± 0.07 | 1.66 ± 0.06 | 0.89 ± 0.08 |
| Replicate 3 | B. raw | 0.0013 | 25.2 ± 2.98 | 9.69 ± 0.35 | *n. d.* | *n. d.* | 3.84 ± 0.16 | 0.08 ± 0.01 | 0.43 ± 0.01 | 3.51 ± 0.14 | 1.35 ± 0.05 | 1.83 ± 0.12 |
|  | B. surface disinfected | 0.0000 | 14.5 ± 0.34 | 9.05 ± 0.39 | *n. d.* | *n. d.* | 2.42 ± 0.11 | 0.09 ± 0.01 | 3.61 ± 0.25 | 127 ± 10.4 | 33.3 ± 3.52 | 0.26 ± 0.01 |
|  | B. 5 days incubated | 0.0100 | 18.9 ± 0.96 | 12.9 ± 0.37 | *n. d.* | *n. d.* | 1.85 ± 0.04 | *< LOQ* | *< LOQ* | 3.81 ± 0.41 | 3.73 ± 0.12 | 0.31 ± 0.00 |
|  | Green malt | 0.0459 | 2.24 ± 0.13 | 5.89 ± 0.23 | *n. d.* | *n. d.* | 0.48 ± 0.02 | *< LOQ* | 0.30 ± 0.02 | 2.88 ± 0.27 | 3.26 ± 0.13 | 0.12 ± 0.01 |
|  | M. with rootlets | 0.0631 | 15.7 ± 0.51 | 13.9 ± 0.68 | *n. d.* | *n. d.* | 1.04 ± 0.04 | *< LOQ* | *< LOQ* | 10.3 ± 1.24 | 5.04 ± 0.10 | 0.49 ± 0.03 |
|  | M. after germ separation | 0.0609 | 27.8 ± 1.22 | 14.1 ± 0.95 | *n. d.* | *n. d.* | 1.17 ± 0.02 | 0.13 ± 0.01 | 2.01 ± 0.05 | 32.3 ± 2.96 | 13.5 ± 1.19 | 0.53 ± 0.03 |
|  | Rootlets | 0.3398 | 6.32 ± 0.36 | 94.0 ± 4.28 | 1.37 ± 0.09 | *< LOQ* | 2.10 ± 0.12 | 0.54 ± 0.01 | 0.95 ± 0.06 | 4.77 ± 0.08 | 2.64 ± 0.14 | 5.37 ± 0.65 |

**Table S5**: Detailed mycotoxin concentrations from inoculation with F. culmorum (10^5^ CFU/mL) at 18 °C across three biological replicates. Data are presented in µg/kg ± SD. All values are given as the mean of triplicate determinations and duplicate injections. Toxins not listed in the table were not detected in any sample. B = barley, M = malt. n. d. = not detected, LOQ = limit of quantification.

|  | **Sample type** | **Fc DNA** | **DON** | **DON-3G** | **3-AcDON** | **15-AcDON** | **HT-2** | **ENN A** | **ENN A1** | **ENN B** | **ENN B1** | **BEA** |
| --- | --- | --- | --- | --- | --- | --- | --- | --- | --- | --- | --- | --- |
|  |  | **pg/ng B. DNA** | **[µg/kg]** | **[µg/kg]** | **[µg/kg]** | **[µg/kg]** | **[µg/kg]** | **[µg/kg]** | **[µg/kg]** | **[µg/kg]** | **[µg/kg]** | **[µg/kg]** |
| Replicate 1 | B. raw | 0.0013 | 25.2 ± 2.98 | 9.69 ± 0.35 | *n. d.* | *n. d.* | 3.84 ± 0.16 | 0.08 ± 0.01 | 0.43 ± 0.01 | 3.51 ± 0.14 | 1.35 ± 0.05 | 1.83 ± 0.12 |
|  | B. surface disinfected | 0.0024 | 20.2 ± 0.97 | 7.57 ± 0.23 | *n. d.* | *n. d.* | 2.56 ± 0.03 | 0.35 ± 0.01 | 4.53 ± 0.21 | 33.2 ± 1.69 | 27.4 ± 3.37 | 0.39 ± 0.02 |
|  | B. 5 days incubated | 0.0011 | 165 ± 1.27 | 8.20 ± 0.30 | 1.38 ± 0.12 | *n. d.* | 1.09 ± 0.04 | 0.29 ± 0.01 | 4.77 ± 0.22 | 127 ± 10.3 | 54.5 ± 1.92 | 0.64 ± 0.02 |
|  | Green malt | 20.123 | 1399 ± 16.4 | 2458 ± 55.5 | 214 ± 23.6 | 6.59 ± 0.19 | 1.01 ± 0.02 | *< LOQ* | *< LOQ* | 0.34 ± 0.00 | 0.12 ± 0.01 | *< LOQ* |
|  | M. with rootlets | 10.461 | 1854 ± 114 | 2826 ± 78.7 | 256 ± 10.8 | 16.5 ± 0.20 | 1.29 ± 0.03 | 0.16 ± 0.01 | *< LOQ* | 1.48 ± 0.03 | 0.47 ± 0.03 | 0.47 ± 0.02 |
|  | M. after germ separation | 6.9915 | 1464 ± 35.9 | 2347 ± 98.1 | 216 ± 3.99 | 13.9 ± 2.38 | 1.60 ± 0.02 | 0.26 ± 0.01 | 1.67 ± 0.13 | 31.6 ± 3.05 | 16.2 ± 1.68 | 0.84 ± 0.11 |
|  | Rootlets | 97.718 | 6074 ± 322 | 6649 ± 272 | 239 ± 19.2 | 18.5 ± 1.79 | 3.14 ± 0.25 | *n. d.* | 0.51 ± 0.07 | 22.5 ± 1.81 | 5.19 ± 0.11 | 1.18 ± 0.03 |
| Replicate 2 | B. raw | 0.0013 | 25.2 ± 2.98 | 9.69 ± 0.35 | *n. d.* | *n. d.* | 3.84 ± 0.16 | 0.08 ± 0.01 | 0.43 ± 0.01 | 3.51 ± 0.14 | 1.35 ± 0.05 | 1.83 ± 0.12 |
|  | B. surface disinfected | 0.0023 | 18.1 ± 0.78 | 7.37 ± 0.19 | *n. d.* | *n. d.* | 2.25 ± 0.07 | 0.10 ± 0.00 | 0.38 ± 0.01 | 2.69 ± 0.11 | 1.51 ± 0.05 | 0.41 ± 0.02 |
|  | B. 5 days incubated | 0.4455 | 20.9 ± 0.42 | 10.3 ± 0.46 | *n. d.* | *n. d.* | 1.45 ± 0.04 | 0.09 ± 0.01 | 0.26 ± 0.01 | 1.96 ± 0.06 | 0.98 ± 0.08 | 0.20 ± 0.00 |
|  | Green malt | 9.8831 | 1952 ± 122 | 2408 ± 193 | 252 ± 11.0 | 8.96 ± 0.28 | 1.76 ± 0.03 | 0.21 ± 0.02 | 2.61 ± 0.25 | 51.9 ± 5.26 | 22.5 ± 1.19 | 0.47 ± 0.01 |
|  | M. with rootlets | 14.361 | 2587 ± 142 | 3136 ± 145 | 362 ± 16.5 | 18.2 ± 1.15 | 3.65 ± 0.06 | 0.13 ± 0.01 | *< LOQ* | 3.20 ± 0.38 | 0.98 ± 0.01 | 0.54 ± 0.01 |
|  | M. after germ separation | 7.7894 | 2421 ± 89.4 | 3532 ± 154 | 421 ± 14.1 | 24.9 ± 3.41 | 1.03 ± 0.02 | 0.20 ± 0.01 | 1.81 ± 0.17 | 99.2 ± 7.98 | 34.5 ± 0.73 | 0.36 ± 0.01 |
|  | Rootlets | 138.19 | 8907 ± 201 | 8407 ± 188 | 264 ± 15.5 | 29.0 ± 3.94 | 0.91 ± 0.01 | *n. d.* | *< LOQ* | 18.4 ± 0.52 | 3.24 ± 0.07 | 0.55 ± 0.00 |
| Replicate 3 | B. raw | 0.0013 | 25.2 ± 2.98 | 9.69 ± 0.35 | *n. d.* | *n. d.* | 3.84 ± 0.16 | 0.08 ± 0.01 | 0.43 ± 0.01 | 3.51 ± 0.14 | 1.35 ± 0.05 | 1.83 ± 0.12 |
|  | B. surface disinfected | 0.0013 | 18.9 ± 0.60 | 7.42 ± 0.20 | *n. d.* | *n. d.* | 2.33 ± 0.13 | 0.10 ± 0.00 | 0.61 ± 0.03 | 6.69 ± 0.50 | 3.31 ± 0.09 | 0.95 ± 0.06 |
|  | B. 5 days incubated | 0.0168 | 20.1 ± 0.39 | 9.95 ± 0.15 | *n. d.* | *n. d.* | 1.93 ± 0.11 | 0.10 ± 0.00 | 0.48 ± 0.02 | 3.17 ± 0.20 | 1.91 ± 0.22 | 0.24 ± 0.01 |
|  | Green malt | 19.326 | 751 ± 36.2 | 1094 ± 78.7 | 64.3 ± 0.59 | 4.68 ± 0.10 | 0.57 ± 0.02 | *< LOQ* | *< LOQ* | 1.04 ± 0.12 | 0.25 ± 0.01 | 0.22 ± 0.02 |
|  | M. with rootlets | 19.386 | 1502 ± 2.96 | 2375 ± 87.6 | 216 ± 3.99 | 12.1 ± 0.49 | 0.54 ± 0.00 | 0.19 ± 0.02 | 1.34 ± 0.13 | 24.4 ± 0.59 | 10.3 ± 0.39 | 0.38 ± 0.00 |
|  | M. after germ separation | 7.8510 | 1543 ± 9.66 | 2323 ± 61.5 | 209 ± 5.31 | 11.9 ± 1.18 | 0.48 ± 0.02 | *n. d.* | *< LOQ* | 12.5 ± 2.12 | 2.90 ± 0.09 | 0.42 ± 0.02 |
|  | Rootlets | 101.93 | 5400 ± 316 | 6491 ± 202 | 211 ± 5.87 | 25.4 ± 1.52 | 1.01 ± 0.13 | *n. d.* | 0.25 ± 0.03 | 18.9 ± 0.47 | 4.61 ± 0.42 | 0.57 ± 0.01 |

**Table S6:** Detailed mycotoxin concentrations from inoculation with F. culmorum (4 x 10^5^ CFU/mL) at 14 °C across three biological replicates. Data are presented in µg/kg ± SD. All values are given as the mean of triplicate determinations and duplicate injections. Toxins not listed in the table were not detected in any sample. B = barley, M = malt. n. d. = not detected, LOQ = limit of quantification.

|  | **Sample type** | **Fc DNA** | **DON** | **DON-3G** | **3-AcDON** | **15-AcDON** | **HT-2** | **ENN A** | **ENN A1** | **ENN B** | **ENN B1** | **BEA** |
| --- | --- | --- | --- | --- | --- | --- | --- | --- | --- | --- | --- | --- |
|  |  | **pg/ng B. DNA** | **[µg/kg]** | **[µg/kg]** | **[µg/kg]** | **[µg/kg]** | **[µg/kg]** | **[µg/kg]** | **[µg/kg]** | **[µg/kg]** | **[µg/kg]** | **[µg/kg]** |
| Replicate 1 | B. raw | 0.0013 | 25.2 ± 2.98 | 9.69 ± 0.35 | *n. d.* | *n. d.* | 3.84 ± 0.16 | 0.08 ± 0.01 | 0.43 ± 0.01 | 3.51 ± 0.14 | 1.35 ± 0.05 | 1.83 ± 0.12 |
|  | B. surface disinfected | 0.0000 | 10.8 ± 0.96 | 7.89 ± 0.28 | *n. d.* | *n. d.* | 4.81 ± 0.34 | 0.11 ± 0.01 | *< LOQ* | 1.27 ± 0.05 | 0.50 ± 0.02 | 1.35 ± 0.12 |
|  | B. 5 days incubated | 35.521 | 11.2 ± 0.38 | 9.68 ± 0.57 | *n. d.* | *n. d.* | 1.12 ± 0.05 | 0.21 ± 0.07 | 1.03 ± 0.09 | 13.5 ± 1.42 | 4.45 ± 0.20 | 0.27 ± 0.02 |
|  | Green malt | 44.752 | 920 ± 95.0 | 2824 ± 303 | 229 ± 21.4 | 10.8 ± 0.26 | 0.92 ± 0.04 | *< LOQ* | *< LOQ* | 2.19 ± 0.06 | 0.22 ± 0.01 | 0.07 ± 0.01 |
|  | M. with rootlets | 31.888 | 1558 ± 136 | 4189 ± 232 | 341 ± 350 | 26.2 ± 0.06 | 1.34 ± 0.01 | *< LOQ* | 0.55 ± 0.03 | 17.5 ± 0.76 | 4.00 ± 0.22 | 0.64 ± 0.03 |
|  | M. after germ separation | 29.821 | 1571 ± 37.7 | 3856 ± 344 | 358 ± 15.2 | 29.4 ± 0.88 | 1.12 ± 0.02 | 0.10 ± 0.00 | 2.37 ± 0.11 | 74.8 ± 8.01 | 18.1 ± 0.91 | 0.37 ± 0.03 |
|  | Rootlets | 5.2282 | 5249 ± 293 | 7585 ± 706 | 357 ± 25.0 | 29.6 ± 0.99 | 4.41 ± 0.19 | 0.82 ± 0.02 | 7.84 ± 0.20 | 26.0 ± 1.23 | 14.4 ± 0.46 | 3.41 ± 0.22 |
| Replicate 2 | B. raw | 0.0013 | 25.2 ± 2.98 | 9.69 ± 0.35 | *n. d.* | *n. d.* | 3.84 ± 0.16 | 0.08 ± 0.01 | 0.43 ± 0.01 | 3.51 ± 0.14 | 1.35 ± 0.05 | 1.83 ± 0.12 |
|  | B. surface disinfected | 1.8183 | 11.9 ± 0.54 | 8.41 ± 0.45 | *n. d.* | *n. d.* | 2.03 ± 0.06 | 0.09 ± 0.01 | 0.89 ± 0.01 | 24.6 ± 0.17 | 7.71 ± 0.11 | 0.23 ± 0.01 |
|  | B. 5 days incubated | 46.118 | 12.3 ± 1.22 | 9.53 ± 0.35 | *n. d.* | *n. d.* | 2.46 ± 0.14 | *< LOQ* | *< LOQ* | 7.45 ± 0.73 | 1.48 ± 0.10 | 0.22 ± 0.02 |
|  | Green malt | 47.317 | 463 ± 66.8 | 1556 ± 89.8 | 125 ± 23.9 | 7.27 ± 0.17 | 0.54 ± 0.02 | *< LOQ* | *< LOQ* | 3.09 ± 0.21 | 0.75 ± 0.02 | *< LOQ* |
|  | M. with rootlets | 27.183 | 920 ± 63.5 | 3148 ± 339 | 170 ± 14.0 | 19.6 ± 1.02 | 1.04 ± 0.02 | *< LOQ* | *< LOQ* | 1.87 ± 0.24 | 0.38 ± 0.03 | 0.35 ± 0.01 |
|  | M. after germ separation | 37.134 | 980 ± 45.9 | 2799 ± 226 | 226 ± 20.5 | 21.2 ± 0.15 | 1.02 ± 0.03 | 0.10 ± 0.00 | 1.69 ± 0.01 | 34.6 ± 2.41 | 8.11 ± 0.25 | 0.43 ± 0.04 |
|  | Rootlets | 4.2344 | 2891 ± 153 | 4932 ± 475 | 234 ± 12.5 | 25.5 ± 0.29 | 2.17 ± 0.11 | 0.26 ± 0.01 | 2.79 ± 0.19 | 11.7 ± 0.83 | 5.50 ± 0.49 | 1.68 ± 0.27 |
| Replicate 3 | B. raw | 0.0013 | 25.2 ± 2.98 | 9.69 ± 0.35 | *n. d.* | *n. d.* | 3.84 ± 0.16 | 0.08 ± 0.01 | 0.43 ± 0.01 | 3.51 ± 0.14 | 1.35 ± 0.05 | 1.83 ± 0.12 |
|  | B. surface disinfected | 0.0003 | 10.5 ± 0.39 | 8.31 ± 0.20 | *n. d.* | *n. d.* | 2.70 ± 0.03 | 0.10 ± 0.01 | *< LOQ* | 1.09 ± 0.03 | 0.31 ± 0.01 | 0.82 ± 0.04 |
|  | B. 5 days incubated | 51.443 | 11.5 ± 0.56 | 9.12 ± 0.13 | *n. d.* | *n. d.* | 2.74 ± 0.16 | *< LOQ* | *< LOQ* | 0.68 ± 0.01 | 0.09 ± 0.00 | 0.84 ± 0.08 |
|  | Green malt | 38.843 | 430 ± 33.5 | 1833 ± 302 | 149 ± 17.7 | 8.83 ± 0.46 | 0.59 ± 0.02 | *< LOQ* | *< LOQ* | 0.59 ± 0.01 | *< LOQ* | 0.40 ± 0.02 |
|  | M. with rootlets | 35.177 | 891 ± 64.0 | 3001 ± 111 | 166 ± 8.03 | 17.9 ± 0.41 | 1.03 ± 0.00 | 0.12 ± 0.00 | 0.83 ± 0.02 | 1.98 ± 0.13 | 1.98 ± 0.13 | 0.39 ± 0.01 |
|  | M. after germ separation | 38.799 | 714 ± 7.18 | 2009 ± 105 | 180 ± 9.53 | 18.2 ± 0.06 | 1.03 ± 0.04 | *< LOQ* | 0.35 ± 0.01 | 2.28 ± 0.05 | 2.28 ± 0.05 | 0.41 ± 0.04 |
|  | Rootlets | 5.8427 | 2516 ± 151 | 4400 ± 330 | 242 ± 8.69 | 25.4 ± 1.47 | 1.54 ± 0.01 | 0.11 ± 0.00 | 1.19 ± 0.08 | 4.42 ± 0.08 | 4.42 ± 0.08 | 2.45 ± 0.00 |
